# Evidence for Gender-Specific Transcriptional Profiles of Nigral Dopamine Neurons in Parkinson Disease

**DOI:** 10.1371/journal.pone.0008856

**Published:** 2010-01-25

**Authors:** Filip Simunovic, Ming Yi, Yulei Wang, Robert Stephens, Kai C. Sonntag

**Affiliations:** 1 Department of Psychiatry, McLean Hospital, Harvard Medical School, Belmont, Massachusetts, United States of America; 2 Bioinformatics Support Group, Advanced Biomedical Computing Center, NCI-Frederick, Frederick, Maryland, United States of America; 3 Applied Biosystems, Foster City, California, United States of America; National Institutes of Health, United States of America

## Abstract

**Background:**

Epidemiological data suggest that the male gender is one of the risks factors for the development of Parkinson Disease (PD). Also, differences in the clinical manifestation and the course of PD have been observed between males and females. However, little is known about the molecular aspects underlying gender-specificity in PD. To address this issue, we determined the gene expression profiles of male and female dopamine (DA) neurons in sporadic PD.

**Methodology/Principal Findings:**

We analyzed Affymetrix-based microarrays on laser microdissected DA neurons from postmortem brains of sporadic PD patients and age-matched controls across genders. Pathway enrichment demonstrated that major cellular pathways involved in PD pathogenesis showed different patterns of deregulation between males and females with more prominent downregulation of genes related to oxidative phosporylation, apoptosis, synaptic transmission and transmission of nerve impulse in the male population. In addition, we found upregulation of gene products for metabolic processes and mitochondrial energy consumption in the age-matched male control neurons. On the single cell level, selected data validation using quantitative Real-Time (qRT)-PCR was consistent with microarray raw data and supported some of the observations from data analysis.

**Conclusions/Significance:**

On the molecular level, our results provide evidence that the expression profiles of aged normal and PD midbrain DA neurons are gender-specific. The observed differences in the expression profiles suggest a disease bias of the male gender, which could be in concordance with clinical observations that the male gender represents a risk factor for sporadic PD. Validation of gene expression by qRT-PCR supported the microarray results, but also pointed to several caveats involved in data interpretation.

## Introduction

Parkinson disease (PD) is a severe neurological disorder of unknown etiology characterized by progressive loss of substantia nigra (SNc) dopaminergic (DA) neurons with the formation of Lewy bodies in the central, peripheral and enteric nervous systems [1]. PD clinically manifests in resting tremor, rigidity, bradykinesia and a wide array of subtler non-motor symptoms such as depression, fatigue, sleep disturbances and autonomic dysfunction.

Aside of age, the male gender could be an additional risk factor for the development of PD as suggested from multiple epidemiological studies [2], [3], [4], [5]. Despite high variability between these studies, it appears that men seem to develop about 1.5 times more sporadic PD than women. This, however, only referred to people in Western populations, since gender did not play a role in PD patients with Asian ethnicity [2], [5].

Additional evidence for a gender effect stems from clinical studies, which reported gender-linked differences in the age of onset and symptomatology of PD. For example, it was found that women developed PD later than men (2.1 years) and presented more frequently with “benign”, tremor-dominant phenotype of the disease, suggesting that at least the initial clinical stages of PD might be milder in the female population [6]. However, other studies found faster disease progression in East Asian women with PD [7] or a difference at the later stages of disease [8], [9]. Despite discrepancies between these reports there appears to be a gender difference in the clinical presentation of PD.

The reasons for variations between genders in PD are not clear, but it is suggested that they are at least in part a consequence of genetic factors [2], [10]. A recent study analyzed the gene expression profile of DA neurons isolated by laser capture microscopy (LCM) from postmortem brains of normal subjects and patients affected with sporadic PD [11]. The authors reported a broad gender-based difference in gene expression that supported the notion that males are predisposed to PD. Similarly, we recently documented the expression profiles of laser microdissected (LMD) SNc DA neurons from 10 PD and 9 control cases and found dysregulation of gene expression in all major pathways relevant to PD pathogenesis [12].

We now extended this study by conducting a comparable gender analysis in our cohort. Altogether, we found differential gene expression in both males and females with distinct patterns of PD-association in each gender. In particular, there was a more prominent downregulation of genes relevant to oxidative phosporylation, apoptosis, synaptic transmission and transmission of nerve impulse in the male population. By comparing the expression profiles of neurons between the normal control groups, we found that males had an enrichment of upregulated genes that are related to many aspects of cellular homeostasis including mitochondrial energy consumption. These data suggest a bias of the male gender towards PD on the molecular level and also could support some of the clinical observations that disease progression and presentation are gender-specific.

## Materials and Methods

### Subjects and Affymetrix-Based Microarrays

A detailed description of the subjects and materials used for this study can be found in our previous study [12] and Table S1. The patients formed the following groups: 3 females in each group of controls and sporadic PD patients, 6 male subjects in the control, and 7 male subjects in the PD group. SNc DA neurons were isolated by LMD using a LEICA AS LMD apparatus, and RNA isolation, amplification and hybridization to the HU-133A arrays (Affymetrix, Santa Clara, CA) were done according to published protocols [13]. Microarray raw data and details about subjects are publicized at the National Brain Databank webpage (http://national_databank.mclean.harvard.edu/brainbank) and partially summarized in Table S1.

### Data Analysis

Data were analyzed using 3-way analysis of variance (ANOVA) and significance analysis of microarrays (SAM) procedure after removing a batch effect as described previously [12]. All differentially expressed genes for the gender analysis were derived from SAM and match the criteria of a false discovery rate (FDR) <5% and p-values <0.01 (stringent conditions) or <0.05 (relaxed conditions). Six different lists were compared for both conditions (details are provided in Tables S2 and S3): all PD patients versus all control cases for ANOVA and SAM (allN_allPD), male control cases versus male PD patients (mN_mPD; mPD), female control cases versus female PD patients (fN_fPD; fPD), control male versus control female cases (mN_fN) and male versus female PD patients (mPD_fPD).

The enrichment-based, pathway-level comparative computational analysis was largely done according to previously published protocols [12]. Briefly, using in-house software WPS and a Pathway Pattern Extraction Pipeline [14] based on a one-sided Fisher exact test, desired gene lists were computed for their enrichment levels using different functional categories including BioCarta pathways, KEGG pathways, or a Gene Ontology (GO-BP) terms. The enrichment levels were then compared amongst these lists in such a way that common or uniquely enriched pathways or terms could be identified. In particular, to test for male- or female-specific PD pathways we established lists that featured only genes that were enriched in mPD or fPD and allN_allPD lists. Based on these lists we created Gene-Term Association Networks (GTAN) that allowed us to further delineate single PD-relevant, male-specific transcripts that are common to two or more major pathways.

In addition to the computational analysis, clustering of probesets was based on our extensive lists of genes previously described as deregulated in PD, and which include a wide spectrum of cellular pathways and processes [12]. This also included a recently published manuscript providing a cross-study analysis of microarray data on PD [15] and available online resources, such as https://ncascr.griffith.edu.au/pdreview/2008/ and www.pdgene.org.

### TaqMan® Real-Time PCR Assay Validation

Validation of gene expression was performed using TaqMan® quantitative Real-Time PCR (qRT-PCR) Gene Expression Assays and the 7900HT Real-Time PCR System (Applied Biosystems, Foster City, CA) for the same 14 genes as described in [12]. The list was extended with three additional male control and PD as well as two female controls and three female PD samples (Table S1). Note that for the male samples C10, C11, PD11 and PD14 and the female sample PD13 no microarray data are available. RNA was isolated from approx. 300 laser-microdissected neurons using the *mir*VANA^TM^ miRNA Isolation Kit (Ambion, Austin, TX) and RNA quality was determined by determining the 260/280 ratios using nanodrop OD measurements (Table S1). cDNAs were generated in a 25 µl reverse transcription reaction with 30–60 ng of total RNA using the High Capacity cDNA Archive Kit and protocol (Applied Biosystems, PN 4322169) and subjected to a 10-cycle PCR amplification followed by qRT-PCR reaction using the manufacturer's TaqMan® PreAmp Master Mix Kit Protocol (Applied Biosystems, PN 4366127). Three replicates per sample were assayed for each gene in a 384-well format plate. For data normalization, GUSB was used as endogenous control gene and gene expression PD versus control was calculated according to the 2^−ΔCt^ method by Livak and Schmittgen [16].

## Results

The analysis of the gene expression profiles followed similar strategies as previously published [12]. Interestingly, gender analysis based on 3-way ANOVA with FDR10 did not reveal differentially expressed genes in fPD. However, we observed striking gender differences using SAM analysis. The SAM list with FDR5 and p-values <0.01 (allN_allPD) was highly overlapping with our previous ANOVA FDR10 list (Figure S1) and reflected the “stringent analysis condition”, whereas the FDR5 list with p-values of <0.05 list was regarded as “relaxed condition”. Altogether, the gender analysis was based on six different gene lists as described in Material and Methods and a “Merged Matrix” (Tables S2 (p<0.01) and S3 (p<0.05)).

### Distribution of Differentially Expressed Genes

**Figure 1:**
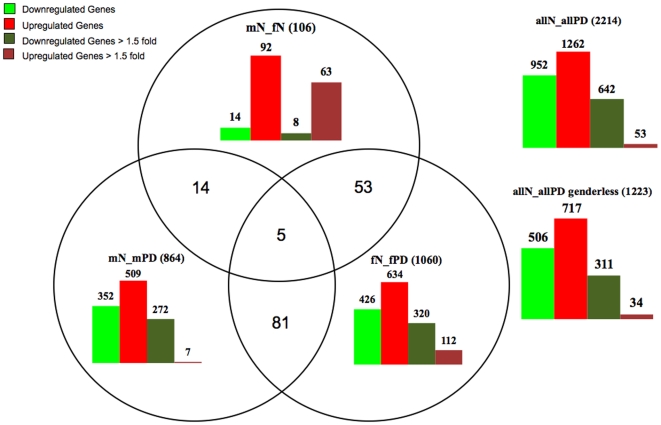
Distribution of gene expression profiles for all groups based on FDR5 p<0.01 analysis. Genes were computationally analyzed (see Material and Methods for details) and summarized schematically. The groups are as follows: allN_allPD compares all normal versus all PD samples; mN_fN compares control males versus control females; mN_mPD compares control males versus male PD; fN_fPD compares control females versus female PD; the overlapping groups indicate the number of genes that are found in several respective groups. Bars represent numbers of up- or downregulated genes in each group for total genes or after setting the cut-off of differential gene expression at >1.5 fold.

**Figure 2:**
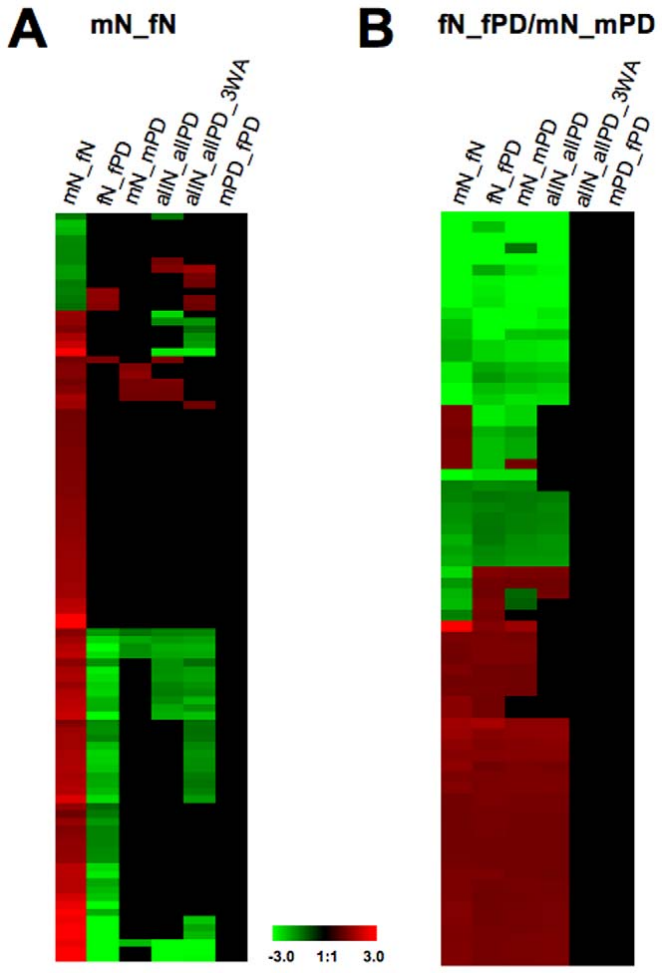
Distribution of overlapping gene expression profiles in normal controls and PD based on FDR5 p<0.01 analysis. (**A**) Heatmaps of those probesets that are expressed in both normal male (mN) and female (fN) DA neurons (first column, mN_fN) in comparison with all other groups (see Table S2 for details). Values shown in the heat maps are log(ratio) with red for upregulated and green for down-regulated genes. A larger subset of the upregulated genes is downregulated in fPD (second column), but not in mPD (fifth column). (**B**) Heatmap of those probesets that are expressed in both female (fN_fPD) and male (mN_mPD) PD DA neurons (first and fourth column) in comparison with all other probesets (see Table S2 for details). The majority of the genes are simultaneously up- or downregulated in both genders, while a small subset of genes is differentially expressed. In addition, levels of gene expression of the overlapping genes are similar in both genders. Note: allN_allPD A3W depicts probesets from the previously published ANOVA FDR10 gene list [12].

The stringent SAM list (FDR5, p<0.01) comprised a total of 2214 deregulated probesets between all control and all PD samples (Table S2, Figure 1) with similar distribution of up- and downregulated genes (952 and 1262, respectively) as seen with ANOVA. Also consistent with ANOVA, we found more downregulated (642) than upregulated (53) genes when the cut-off was set at >1.5 fold difference, with downregulation reaching as high as 11 fold, while only six genes exceeded a 2 fold upregulation (the maximum being at 3.4 fold). The distribution of differentially expressed genes within and across the gender groups showed more up- than downregulation. However, when the cut-off was set at >1.5 fold difference, all groups except of mN_fN had more genes down- than upregulated. This was especially obvious for mPD, in which only seven from 509 genes were >1.5 fold upregulated, in contrast to 112 out of 634 genes in fPD (four genes with >3.0 fold). Interestingly, we observed only a moderate downregulation (14 probesets with 8>1.5 fold), but a striking upregulation of genes (92 probesets, 63>1.5 fold) in normal control neurons from males when compared to females (mN_fN) and the majority of these genes were downregulated in fPD (Figure 2A).

Analysis of gene overlaps between the groups revealed that 5 genes were present in all groups, 14 genes overlapped between mPD and mN_fN, 53 genes overlapped between fPD and mN_fN, and 81 probesets (3.7% of all deregulated genes) were present in both mN_mPD and fN_fPD (Figure 1, Table S2). When we directly compared the probesets between the mPD and fPD lists, we found only four overlapping genes (Table S2). Thus, the overwhelming number of genes was dysregulated in either females or males, while only a relatively small number overlapped between both groups. Interestingly, the majority of the gender-overlapping genes were simultaneously up- or downregulated in males and females, while only a small subset of genes was differentially expressed (Figure 2B, Table S2). Moreover, just 13 of these probesets related to PD pathogenic pathways and the majority of them were downregulated (Table 1). It should be noted that from the allN_allPD group we found 1223 differentially expressed genes that did not appear in either gender and some of them were part of gene clusters in all the PD-associated pathways (Figure 1, Table S2). Taken together, these data indicate that dysregulated gene expression seems to be gender-specific.

**Table 1:** PD-associated genes present in fPD and mPD (FDR5, p<0.01).

Gene Symbol	GenBank ID	Description	allN_allPD	fPD	mPD	mN_fN	mPD_fPD
ATP5G3	NM_001689	ATP synthase H+ transporting, mitochondrial F0 complex subunit C3	−2.25	−2.17	−2.94	0	0
ATP6V1E1	NM_001696	ATPase, H+ transporting, lysosomal 31kDa, V1 subunit E1	−3.57	−4.76	−3.23	0	0
CHRNA4	NM_000744	cholinergic receptor, nicotinic, alpha 4	1.25	1.4	1.22	0	0
CLTC	NM_004859	clathrin, heavy chain (Hc)	−1.33	−5.56	−3.12	0	0
COX7C	NM_001867	cytochrome c oxidase subunit VIIc	−2.90	−2.56	−3.64	0	0
DRD2	NM_016574	dopamine receptor D2	1.26	1.33	1.32	0	0
NDUFB2	NM_004546	NADH dehydrogenase (ubiquinone) 1 beta subcomplex 2	−3.39	−2.22	−3.70	0	0
PRKACB	NM_207578	protein kinase cAMP-dependent catalytic beta	−2.44	1.35	−2.70	0	0
SLC35A1	NM_006416	solute carrier family 35 (CMP-sialic acid transporter) member A1	−1.49	−1.79	−1.37	0	0
SLC6A1	NM_003042	solute carrier family 6 (neurotransmitter transporter GABA), member 1	−1.67	−1.85	−1.56	0	0
TIMM44	NM_006351	translocase of inner mitochondrial membrane 44 homolog (yeast)	0	−1.49	1.26	0	0
UQCRH	NM_006004	ubiquinol-cytochrome c reductase hinge protein	−2.94	−3.45	−2.63	0	0
ZNF606	NM_025027	zinc finger protein 606	1.31	1.4	1.27	0	0

We also analyzed gene distributions in the SAM FDR5 p<0.05 gene lists. As expected, in most groups the overall number of deregulated probesets increased, but the distributions of genes largely remained the same (details are provided in Figure S2 and Table S3). Interestingly, there was no major change in numbers of deregulated genes in the mN_fN group, while the amount of probesets that did not appear in either gender decreased about 2-fold (allN_allPD genderless). Also, the number of probesets that were present in both mN_mPD and fN_fPD increased unproportionally to 610 (18% of all deregulated genes). Despite these differences, the more relaxed SAM analysis also pointed to gender-specific deregulation of gene expression.

### Gender-Specific Gene Expression in Normal DA Neurons

Prior to the analysis of gene expression profiles related to PD, we investigated whether gender-specific gene expression occurred in normal DA neurons. The most prominent observation was that, relative to females, in normal male neurons only 14 genes were downregulated, while 92 genes were upregulated (Figure 1, Figure 2A, Table S2). GO-BP enrichment analysis revealed that these upregulated genes are involved in many aspects of cellular homeostasis (Table S4) that also included some key molecules related to mitochondrial processes, such as glutathion S-transferase theta 1 (GSTT1), potassium intermediate/small conductance calcium-activated channels (KCNN3), NADH dehydrogenase (ubiquinone) 1 subcomplex (NDUFC1), S100 calcium binding protein A1 (S100A1), and solute carrier family 11 (proton-coupled divalent metal ion transporters) member 2 (SLC11A2). The majority of the upregulated genes were simultaneously downregulated in female PD alone (Figure 2A, Table 2), including several genes that are associated with pathways related to PD pathogenesis such as ST13, MAPT, XPO1, SLC11A2, DNAJC7, KCNN3, APBA2 and TNS1. These genes were also clustered in the 53 probesets that overlapped in the mN_fN and fPD gene lists (Figure 1). In contrast, the five genes present in all three gene lists and those that overlapped within the mN_fN and mPD groups (Figure 1, Table S2) did not seem to have any obvious PD-associated functions.

**Table 2:** PD-associated genes present in mN_fN (FDR5, p<0.01).

Gene Symbol	GenBank ID	Description	allN_allPD	fPD	mPD	mN_fN	mPD_fPD
AKAP8L	NM_014371	A kinase (PRKA) anchor protein 8-like	0	1.54	0	-1.41	0
APBA2	NM_005503	amyloid beta (A4) precursor protein-binding, family A, member 2 (X11-like)	−3.23	−7.69	0	3.14	0
DDX11	XM_001124814	DEAD/H box polypeptide 11	1.31	0	1.27	1.26	0
DIDO1	NM_080797	death inducer-obliterator 1	0	0	0	1.25	0
DNAJC7	NM_003315	DnaJ (Hsp40) homolog subfamily C member 7	−1.67	−2.70	0	1.77	0
DNM2	NM_004945	dynamin 2	0	0	0	1.36	0
FOXO3	NM_201559	forkhead box O3	0	−3.45	0	2.93	0
GSTT1	NM_000853	glutathione S-transferase theta 1	0	−1.61	0	1.61	0
KCNN3	NM_170782	potassium intermediate/small conductance calcium-activated channel subfamily N	0	−1.92	0	1.93	0
MAPK6	NM_002748	mitogen-activated protein kinase 6	0	−2.56	0	2.38	0
MAPT	NM_016841	microtubule-associated protein tau	0	−5	0	3.15	0
MICAL2	NM_014632	microtubule associated monoxygenase calponin and LIM domain containing 2	0	0	0	1.23	0
NDUFC1	NM_002494	NADH dehydrogenase (ubiquinone) 1 subcomplex unknown 1	−1.72	−2.56	0	1.88	0
PPP2R5E	NM_006246	protein phosphatase 2 regulatory subunit B' epsilon isoform	0	0	0	1.40	0
S100A1	NM_006271	S100 calcium binding protein A1	0	0	0	1.49	0
SLC11A2	NM_000617	solute carrier family 11 (proton-coupled divalent metal ion transporters) member 2	0	0	0	1.68	0
SLC35E3	NM_018656	solute carrier family 35 member E3	0	0	0	−1.56	0
SOX4	NM_003107	SRY (sex determining region Y)-box 4	1.42	0	0	−1.78	0
ST13	NM_003932	suppression of tumorigenicity 13 (Hsp70 interacting protein)	−1.67	−1.67	0	1.52	0
STX8	NM_004853	syntaxin 8	0	−1.96	0	1.88	0
TNS1	NM_022648	tensin 1	−4.54	−6.25	0	3.99	0
TSPAN31	NM_005981	tetraspanin 31	0	0	0	1.35	0
XPO1	NM_003400	exportin 1 (CRM1 homolog, yeast)	−1.51	−1.78	1.46	−1.33	0
ZER1	NM_006336	zer-1 homolog (C. elegans)	0	−1.75	0	1.60	0

### Enrichment-Based Pathway-Level Comparative Analysis

**Figure 3:**
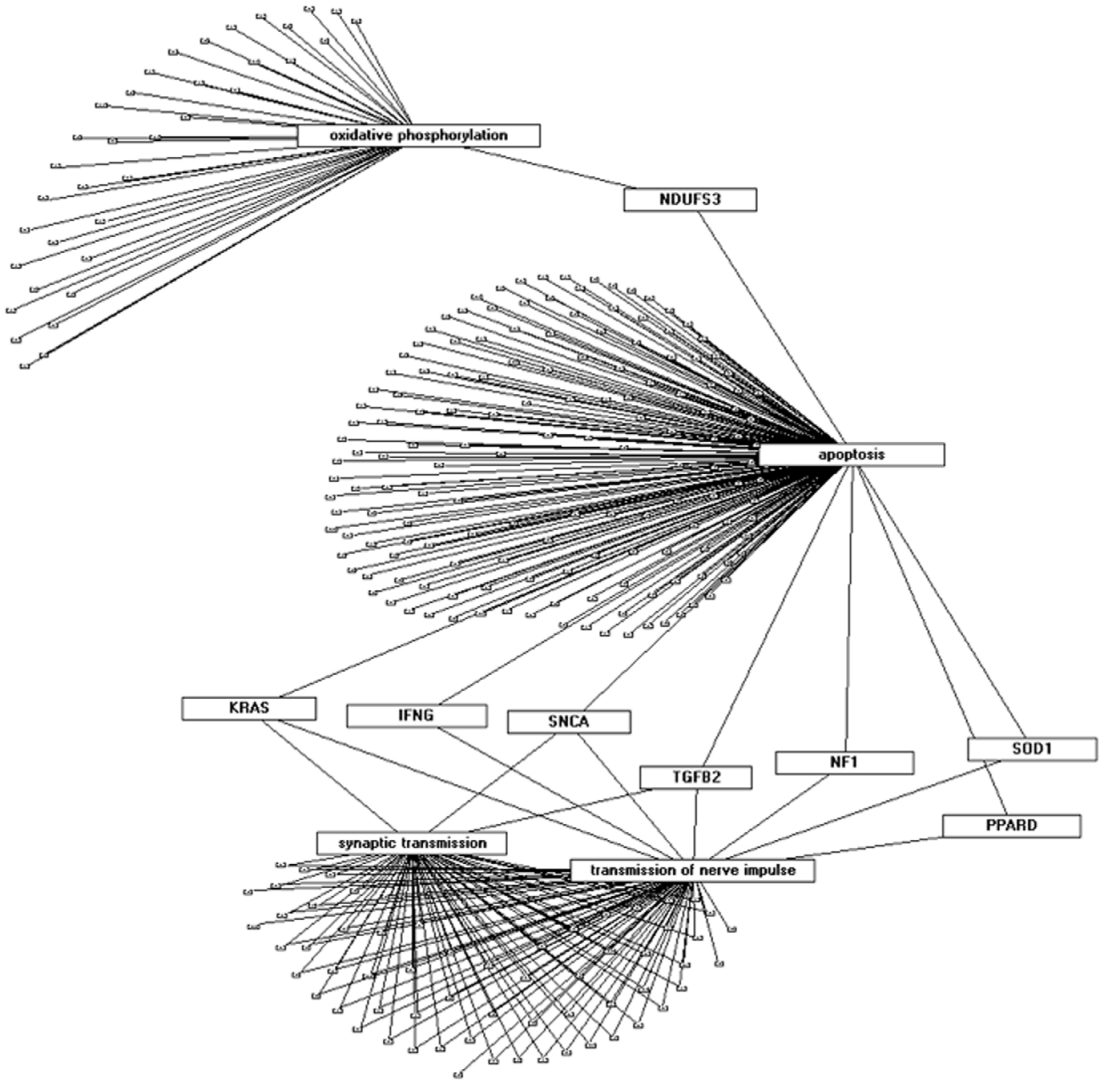
Gene-Term Association Network (GTAN) of selected GO-BP terms (oxidative phosphorylation, apoptosis, synaptic transmission, transmission of nerve impulse) in a population of genes that are deregulated in both allN_allPD SAM and allN_allPD 3-Way ANOVA groups, as well as mPD based on FDR5 p<0.01 analysis. Shown are paramount PD-related transcripts that overlap between the terms.

We next used enrichment-based pathway-level comparative analysis [12], [14] to cluster genes according to pathways of biological function. In all analyses (Biocarta, KEGG, GO-BP, GSEA) we observed that female and male transcripts clustered around specific terms (Figures S3, S4, and S5), which prompted us to generate enrichment levels only for those terms that are enriched in allN_allPD and mPD or allN_allPD and fPD lists (Figures S4 and S5). In both GO-BP and GSEA analysis we found a prominent enrichment of terms in mPD, but not in fPD. In addition, when we used the GO-BP terms to establish Gene-Term Association Networks pinpointing genes that are enriched in pathways relevant to PD, we found a set of genes in mPD that were associated with two or more of the following terms: Oxidative phosphorylation, apoptosis, synaptic transmission, and transmission of nerve impulse (Figure 3). These results indicated a bias of some key molecules in PD pathogenesis, such as SNCA, SOD1, TGFB2, IFNG, NDUFS3, NF1, PPARD, and KRAS towards the male gender. Similar results were also obtained when the gene lists were analyzed under the more relaxed criteria (p<0.05) (Figure S6). When comparing the data from both stringent and relaxed conditions there was an increased enrichment of terms in both mPD and fPD with higher variation in the female group and more consistency with allN_allPD in the male PD group (Figure S6a). In all analyses, there was a consistent strong enrichment of pathways relevant to PD pathogenesis including oxidative phosphorylation, synaptic transmission, and transmission of nerve impulse in mPD (Figure S6b – d).

### Gender-Specific Gene Expression in PD DA Neurons

**Figure 4:**
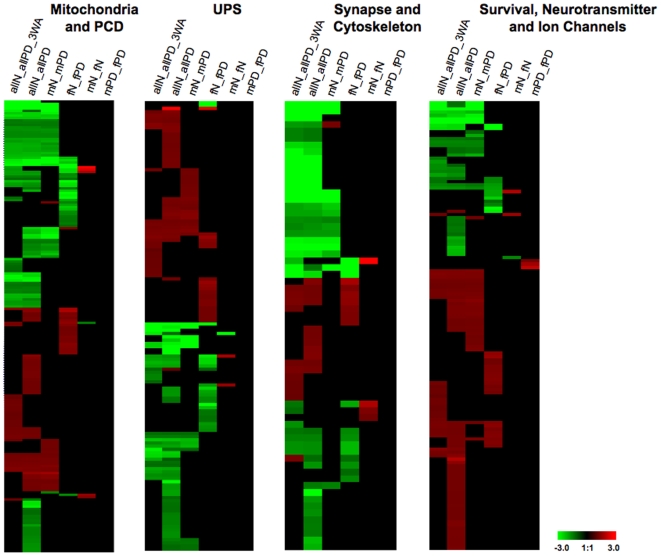
Gender-specific expression profiles of probesets relevant to pathways in PD based on FDR5 p<0.01 analysis. Heatmaps showing up- or downregulated genes in female (mN_fPD) and male (mN_mPD) PD compared to all other groups. In all pathways, the overwhelming number of genes was dysregulated in either gender with little overlap between both groups. Note: allN_allPD A3W depicts probesets from the ANOVA FDR10 gene list previously published [12].

Although the comparative pathway-level analysis demonstrated a dominant PD-specific enrichment only in mPD, closer inspection of the gene lists showed a more complex picture. Assessment of the lists according to those pathways that directly correlate with PD pathogenesis (Table S5) revealed that in all pathways gene groups were dysregulated in both mPD and fPD (Figure 4). Interestingly however, and consistent with the data for all probesets (Figure 1, Table S2), the majority of these genes were specific to either male or female PD and there was little overlap between both genders. These results further indicated that deregulated gene expression was gender-specific. To confirm this observation, we additionally analyzed our data set by focusing on single genes or clusters of genes similarly to our previous report [12] taking in account both the stringent (FDR5 p<0.01) and the relaxed (FDR5 p<0.05) analysis criteria.

### PARK Genes

A prominent finding in the stringent data analysis was downregulation of several genes from the PARK gene family in male PD alone (Table 3), such as PARK1 (SNCA), PARK6 (PINK1), and PARK7 (DJ-1), which are linked to familial forms of the disease. However, when the data were analyzed under the more relaxed conditions, only PARK7 together with UCHL1 were down-regulated in the males alone, while downregulation of ATP13A2 and RIMS3 was restricted to females. Interestingly, in both analysis conditions there were no disease or gender-specific differences in the expression of Parkin.

**Table 3:** Genes associated with PD linkage (PARK loci).

PARK	Gene Symbol	GenBank ID	Description	All^1^	Males p<0.01	Males p<0.05	Females p<0.01	Females p<0.05
PARK1	SNCA	BG260394	synuclein alpha	−1.97	−2.12	−1.13		−1.8
PARK2	Parkin	NM_004562	PD (autosomal recessive, juvenile) 2					
PARK5	UCH-L1	NM_004181	ubiquitin carboxyl-terminal esterase L1	−1.88		−1.72		
	HIP2	NM_005339	huntingtin interacting protein 2	−1.21	1.20^2^	1.2		
PARK6	PINK1	AF316873	PTEN induced putative kinase 1	−2.06	−1.83	−1.83		−2.56
PARK7	DJ-1	NM_007262	PD (autosomal recessive early onset) 7	−7.08	−9.09	−8.37		
PARK9	ATP13A2	NM_022089	ATPase type 13A2	−1.34				−1.64
PARK10	RAP1GAP	AB007943	RAP1 GTPase activating protein	1.40	1.36	1.36		1.47
	RIMS1	AF263310	reg. synaptic membrane exocytosis 1	1.20				
	RIMS3	NM_014747	reg. synaptic membrane exocytosis 3	−2.51			−5.47	−5.47
PARK13	HTRA2		mitochondrial serine protease	−1.54^3^		−1.33		

1 data combined for p<0.01 and p<0.05.

2 HIP1 (U79734).

3 HTRA1 (NM_002775).

### Deregulation of Cellular Compartments

The microarrays revealed a complex picture of gene deregulation associated with extrinsic and intrinsic forms of apoptosis, mitochondrial function, protein degradation pathways, synapse and cytoskeleton, as well as growth factors, neurotransmitter receptors and ion channels (Table S5). According to our previous observations, there was an upregulation of genes involved in signaling pathways related to extrinsic apoptosis and this was particularly prominent in fPD. A more complex picture was observed for genes related to mitochondrial function with a mixed pattern of mostly downregulated gene expression in both female and male PD. Noticeable differences could be observed for protein phosphatase and kinase subunits that were upregulated in females and a more pronounced and higher downregulation of ATP synthase/H+ transporting, cytochrome c oxidase, and NADH dehydrogenase subunits in male PD. For the ubiquitin proteasome system we also observed a mixed picture of up- and downregulated gene groups in both genders. Noticeable differences could be seen for heatshock and ubiquitin-associated proteins that were more downregulated in either mPD or fPD, respectively. Interestingly, in both groups, there was a prominent upregulation of Zink finger proteins, although without a clear overlapping pattern between the genders. Differences of gene expression related to synaptic function and cytoskeleton could be observed for all gene groups between both genders. Consistent with our previous report [12], there was an overall upregulation of genes related to cell survival but without obvious difference between mPD and fPD. In the stringent conditions we found a more prominent downregulation of GABA receptor subunits and associated proteins in mPD, while in both genders gene groups for glutamate, cholinergic, somatostatin and dopaminergic receptors were upregulated. For the ion-channel related transcripts, there was a mixed picture of up- and downregulated genes in both groups without a clear bias to either gender.

### Gender-Specific Key Genes in PD

Our computational data set indicated that deregulated gene expression in PD is gender-specific with a bias of some key genes towards the male gender. To further corroborate this finding, we composed a list of genes that fulfilled the following criteria: 1. Enrichment in pathway-level comparative analysis (see above); 2. Association with PD pathogenesis; 3. Other previously published microarray studies on PD (summarized in [15], https://ncascr.griffith.edu.au/pdreview/2008/); and 4. Overlap with genetic association studies on PD phenotypes (www.pdgene.org) and data from four main PD Genome-Wide Association Studies (GWAS) [17], [18], [19], [20], [21]. In both the stringent and relaxed data analysis, this list of 36 key genes (Table 4) contained 15 probesets that were deregulated in mPD, 10 in fPD and only 2 that overlapped between both genders, while 9 probesets changed when the p-values were shifted from <0.01 to <0.05. This result is largely consistent with the data from the pathway-enrichment and gene cluster analyses indicating a gender-specific dysregulation of gene expression in PD-affected DA neurons with a possible bias towards the male gender.

**Table 4:** Key genes associated with PD.

Gene Symbol	GenBank ID	Description	All^1^ p<0.01	Males p<0.01	Males p<0.05	Females p<0.01	Females p<0.05
***Genes associated with PCD and mitochondrial function***							
TNFRSF1A	NM_001065	tumor necrosis factor receptor 1A	0	0	0	1.25	1.26
PINK1	NM_032409	PTEN induced putative kinase 1	−2.08	−1.85	−1.82	0	−2.55
SOD1	NM_000454	superoxide dismutase 1, soluble	−3.44	−3.44	−3.44	0	0
PARK7	NM_007262	Parkinson disease 7	−7.14	−9.09	−8.37	0	0
NDUFA6	NM_002490	NADH dehydrogenase 6, 14 kDa	−1.80	0	−2.19	−1.56	−2.07
NDUFB8	NM_005004	NADH dehydrogenase 8, 19 kDa	−7.14	−5.55	−3.00	0	−10.96
CYP1A1	NM_000499	cytochrome P450, family 1, subfamily A	1.2	0	0	1.36	1.36
CYP2C9	NM_000771	cytochrome P450, family 2,subfamily C	0	1.23	1.23	0	0
APAF1	NM_181869	apoptotic peptidase activating 1	1.35	1.41	1.40	0	0
GSTA4	NM_001512	glutathione S-transferase A4	−1.38	−1.38	−1.38	0	0
***Genes associated with protein degradation***							
SNCA	NM_007308	synuclein, alpha	−1.88	−2.12	−1.12	0	−1.84
PSMA7	NM_002792	proteasome alpha, 7	−1.72	0	0	−1.78	−1.77
HSP90AA1	NM_005348	heat shock protein 90 kDa alpha	−2.70	−3.33	−3.46	0	0
HSPA8	NM_153201	heat shock 70 kDa protein 8	−2.77	−3.70	−1.35	0	0
LAMP1	NM_005561	lysosomal-associated membrane protein 1	0	0	0	−12.50	−11.50
PPARD	NM_006238	peroxisome proliferator-activated receptor δ	0	1.20	1.20	0	0
***Growth factors, receptors and ion channels***							
DRD1	NM_000794	dopamine receptor D1	0	0	0	1.37	1.37
DRD2	NM_016574	dopamine receptor D2	1.26	1.32	1.32	1.32	1.32
CHRNA4	NM_000744	cholinergic receptor, nicotinic,alpha 4	1.25	1.22	1.22	1.4	1.40
GABARAPL2	NM_007285	GABA(A) receptor-associated protein-like 2	−1.53	−1.51	−1.50	0	−1.54
GRIN2B	NM_000834	glutamate receptor, NMDA 2B	1.27	1.27	1.27	0	1.25
TGFB2	NM_003238	transforming growth factor, beta 2	1.24	0	1.24	1.33	1.33
KCNN3	NM_170782	potassium intermediate/small conductance Ca-activating channel, subfamily N, member 3	0	0	0	−1.92	−1.91
SLC24A3	NM_020689	solute carrier family 24 (Na/K/Ca exchanger)	0	−1.56	−1.55	0	0
SLC6A4	NM_001045	solute carrier family 6 (serotonin)	0	0	0	1.33	1.33
CCK	NM_000729	cholecystokinin	−1.23	−1.31	−1.30	0	0
CCKAR	NM_000730	cholecystokinin A receptor	1.31	1.31	1.31	0	0
VDR	NM_001017535	vitamin D receptor	1.39	1.39	1.39	0	1.31
FYN	NM_153048	FYN oncogene related to SRC, FGR, YES	−1.19	−1.19	−1.19	0	−1.92
RIPK2	NM_003821	receptor-interacting serine-threonine kinase 2	1.29	0	0	1.29	1.29
***Other***							
MAPT	NM_016841	microtubule-associated protein tau	−5	0	0	−5	−4.9
FTH1	NM_002032	ferritin, heavy polypeptide 1	−2.81	−2.77	−2.74	0	0
FTL	NM_000146	ferritin, light polypeptide	−1.88	−1.81	−1.80	0	0
PPP1R3A	NM_002711	protein phosphatase 1, regulatory (inhibitor) 3A	1.25	1.24	1.24	0	0
PPP3CA	NM_000944	protein phosphatase 3 alpha	−2.56	−3.03	−2.94	0	0
ST13	NM_003932	supp. of tumorigenicity 13 (Hsp70 inter. prot.)	−1.67	0	0	−1.64	−1.67

### Validation of Microarray Data Using TaqMan®-Based Real-Time PCR

**Figure 5:**
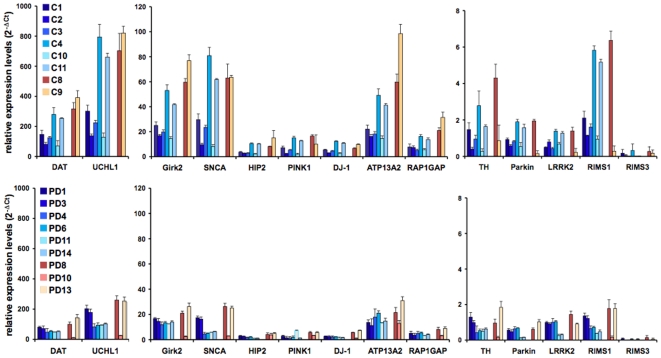
Validation of gene expression by TaqMan® qRT-PCR. Data were analyzed using the 2^−ΔCt^ method [16] and plotted as relative expression levels of individual genes when normalized to GUSB as internal controls. The bars depict results from six individual male (C1, C2, C3, C4, C10, C11) and two female controls (C8, C9) as well as six male PD (PD1, PD3, PD4, PD6, PD11, PD14) and three female PD samples (PD8, PD10, PD13) (Table S1). Note that samples C3, C10, C11, PD3, PD4, and PD11 were taken from [12]. Genes were clustered according to high (DAT, UCHL1), medium (Girk2, SNCA, HIP2, DJ-1, ATP13A2, RAP1GA1), and low (TH, Parkin, PINK1, LRRK2, RIMS1 and 3) expressing genes.

Finally, we validated some of the microarray results using TaqMan®-based qRT-PCR on laser-microdissected neurons according to our previous study [12]. For data consistency and because of the differences in PARK gene expression (see above), we used the same qRT-PCR array amplifying the gene products for tyrosine hydroxylase (TH), dopamine transporter (DAT or SNC6A3), Girk2 (KCNJ6) and all PARK genes (Table 3) including LRKK2, which was not present on the HG-U133A Affymetrix chip. In the male groups, three additional samples were added for controls and PD, respectively. In the female group we included two control and three PD samples. Data were analyzed according to the 2^−ΔCt^ method [16] using GUSB as internal control for normalization. These experiments largely confirmed our previous results demonstrating a considerable variation in the expression of individual genes (up to 2 logs and regardless of disease or gender) and across the sample population (Figure 5). In addition, there was overall lower gene expression in the PD samples and, to our surprise, also for most of the PARK genes. To corroborate these findings, we additionally plotted the results as heat maps and scatter plots (Figure S7). On the individual sample level, there was high consistency between the normalized values in z-scores and the 2^−ΔCt^ values and a general down-regulation of gene expression in the PD samples (Figure S7a-e). It should be noted that in the male PD population two probesets for SNCA showed up- and two probesets down-regulation in the z-scores (Figure S7a and d). Also, in both female and male PD, the z-scores for Parkin were upregulated (Figure S7a and e), although this result seemed to be exaggerated, since the difference from the mean expression level was very small (Figure S7b). Finally, we compared the qRT-PCR results with the data points from the SAM analyses. After data transformation, we found that there was higher correlation with the values derived from the relaxed (FDR5 p<0.05) than with the stringent criteria (FDR5 p<0.01) (Table 3).

## Discussion

Our study demonstrates that deregulated gene expression in PD follows different patterns between genders with indication of a male bias towards the disease. These results are somewhat in line with studies on patient populations concluding that the male gender could represent a risk factor for the development of sporadic PD [2], [3], [4], [5] and could also support clinical observations on gender-specific disease development and progression [6], [7]. Based on a multi-leveled approach using PD-relevant pathway analysis [12], computational and gene-term association networks as well as comparative data mining from published literature on PD pathogenesis, gender effects, and PD-associated gene lists, we identified a set of genes that we suggest as primary factors in PD and gender specificity.

### Gender-Specific Expression Profiles in Aged Control DA Neurons and Their Implication on PD

Epidemiological studies suggested that gender- and race-related genetic factors could influence the function of DA neurons and, consequently, the risk for PD. In control age-matched DA neurons, we observed 106 transcripts that were differentially expressed between females and males with 92 of them higher expressed in males. Many of these genes were involved in multiple aspects of cellular homeostasis and also included some key molecules in energy-consuming mitochondrial processes, such GSTT1, KCNN3, NDUFC1, S100A1, and SLC11A2 suggesting a male-specific increase in mitochondrial activity and Ca^2+^ homeostasis. Since these and several other upregulated transcripts, such as APBA2, DNAJC7, DNM2, MAPT, ST13 and TNS1 are linked to PD pathogenesis, our data could support previous suggestions that a high metabolic rate of DA neurons might accelerate their ageing and predisposition to PD [22]. Thus, this conclusion would imply that levels of gene expression are not only a characteristic of disease state, but could also be a factor in disease development. Indeed, our qPCR analysis demonstrated that gene expression differs across normal control subjects and that expression levels of different genes substantially vary (>2 logs) within the same LMD DA neuron pool. It appears therefore that a combination of gene expression levels, individual predisposition (e.g. genetic variability [23]), and gender could influence the vulnerability or protection of DA neurons towards the development of PD.

### Gene Expression Profiles in Late-Stage PD DA Neurons

In our study the overwhelming number of deregulated genes did not overlap between females and males. In fact, only 3.7% or 18% of the deregulated transcripts were up- or downregulated in both genders using the stringent (p<0.01) or relaxed (p<0.05) analysis criteria, respectively. This group included transcripts relevant to PD cellular pathways, such as mitochondrial function, synaptic transmission, neurotransmitter and ion channel activity, and therefore, could represent a set of disease-driving genes that are common to both male and female PD (e.g. CHRNA4, CLTC, COX7C, DRD2 and NDUFB2). However, regardless of these common transcripts, the high divergence between the genders indicated that the expression profiles of PD-affected DA neurons seem to be gender-specific. This was also evident when we clustered the data according to the established signaling pathways linked to PD [12]. Interestingly, this analysis did not demonstrate an obvious PD-linked biological divergence between both groups, i.e., neither profile pointed to a particular or prominent gender-specific dysfunction of PD-relevant pathways. Rather, there was a marked divergence amongst subtypes of single gene families, which could have been the driving force in the pathway enrichments resulting in the observed male bias towards PD.

A gene group of special interest refers to the PARK genes, which have been linked to familial forms of PD [24], [25] and our previous data analysis [12] together with results from other studies [26], [27], [28], [29] have provided evidence that these molecules are also involved in idiopathic PD. Our results from the gender analysis revealed that SNCA, DJ-1 and PINK1 were exclusively down-regulated in male PD under the stringent (FDR5, p<0.01) analysis criteria. However, both the relaxed conditions (FDR5, p<0.05) and the qRT-PCR experiments showed a general trend towards similar down-regulation of all PARK genes in both genders, although this was less evident for DJ-1. Differences between data sets could be due to multiple factors. For example, all PARK genes except of UCHL1 were relatively low expressing genes raising the question of how significant differential gene expression profiles in microarray studies are. It is common that low expressing genes in microarrays are usually difficult to validate and can often be filtered out from the analysis. Here, we did not filter these terms due to their claimed importance in PD pathogenesis. In addition, in male PD two probes for SNCA revealed up- and two probes downregulation by z-scores pointing to an even more complex level of assessing gene expression that relates to the detection of different splice forms. Nevertheless, our extended qPCR analysis together with the microarrays demonstrated a more general down-regulation of PARK in PD. This has implication for disease pathogenesis, since the PARK proteins influence several key pathways of cellular function including oxidative phosphorylation, apoptosis, synaptic transmission, and transmission of nerve impulse, all pathways that seem to be more affected in male PD.

We eventually evaluated the gene expression profiles combining different criteria, such as PD-relevant pathway analysis as previously described [12], computational gene-term association networking, and comparative data mining from published literature on PD pathogenesis and PD-associated gene lists. These analyses in association with gender-specificity identified a set of genes that could be primary factors in PD and could be involved in a predisposition of males towards this disorder (Table 4). A prominent group of deregulated genes referred to PCD and mitochondrial dysfunction, such as DJ-1 (PARK7), which serves as a redox-sensitive chaperone in oxidative stress response [30]. Its mutation causes recessive early-onset PD [31]. Recently, it has been shown that DJ-1 forms a ubiquitin E3 ligase complex together with Parkin and the serine-threonin mitochondrial kinase PINK1 and promotes the degradation of miss-folded proteins [32]. DJ-1 and PINK1 were downregulated in both genders with a trend towards the male population. Interestingly, there seems to be a connection between PINK1 and the TNF pathway, as one of PINK1's substrates is the TNF-receptor associated protein 1 (TRAP1) [33], which was downregulated in male PD. In addition, there was deregulation of several other genes linked to TNF pathways specific to both males and females and from the group of primary factors, we found a 1.25-fold upregulation of the TNF receptor 1A in the female population. These data indicate that molecules related to extrinsic apoptosis might also contribute to gender-specificity in PD pathogenesis. Another markedly downregulated “male-specific” gene related to oxidative stress response was SOD1, which is primarily associated with amyotrophic lateral sclerosis (ALS), but common pathways with PD have been suggested [34]. A reduction in its activity has been reported as a commonality between PD, Alzheimer's disease, ALS and Huntington's disease [35]. The list of genes also included several members of the mitochondrial electron transport chain like complex I-associated NADH dehydrogenase, cytochrome P450, and glutathione S-transferase family members, which are potentially important in PD pathogenesis [36], [37], [38], [39], [40]. These genes are deregulated in both genders with a trend towards a male bias. PCD is also associated with disruption of the UPS and mechanisms of protein degradation [41]. From the key genes related to this PD-associated pathway, the heat shock family members HSP90AA1 and HSPA8 are downregulated in males, while the proteasome alpha subunit 7 (PSMA7) is downregulated in females, which could indicate a “stronger” impact of the disease on disrupting metabolic processes in the male population. Interestingly, females exhibited marked downregulation of lysosomal-associated membrane protein 1 (LAMP1), which is associated with the function of phagosomes, endosomes and lysosomes. For example, this molecule has been linked to impaired endosome and phagosome movement by disrupting dyneins [42], [43] implicating its association with molecules of the cytoskeleton and its potential role in disrupting cytoskeletal cell function in PD.

It is commonly established that the dynamics of growth factor function, neurotransmitter receptor and ion channel activity in nigral dopaminergic neurons are an important aspect of PD pathogenesis [22], [44], [45], [46], [47], [48]. In agreement with our previous study, the list of PD-associated primary factors contains several of them that are dysregulated in both females and males, but predominance in the male population was less evident. In particular, we observed an upregulation of the dopamine receptors 1 and 2, cholinergic receptor alpha 4, glutamate receptor 2, and transforming growth factor beta 2, while the transcript for GABA(A) receptor associated protein was downregulated. An interesting observation was the upregulation of the vitamin D receptor (VDR) and downregulation of ferritin light polypeptide (FTL) and ferritin heavy polypeptide 1 (FTH1) with some bias towards the male population. Vitamin D deficiency has been linked to an increased incidence of PD [49] and there is evidence that iron metabolism is associated with PD pathogenesis [50], [51], [52], [53]. Another set of genes relevant to PD pathogenesis is cholecystokinin (CCK) and its receptors (CCKAR), which are down- and upregulated in males, respectively. Cholecystokinin is involved in modulating dopamine in the mesolimbic pathway and implicated in the psychiatric symptomatology of PD. A recent gene-linkage study supports the notion that CCK and CCKR polymorphism might be associated with hallucinations in PD [54]. Finally, the list of genes contains suppression of tumorigenicity 13 (ST13) and microtubule-associated protein tau (MAPT). ST13 is part of a number of marker genes that were recently proposed as possible biomarkers in PD [55] and MAPT is linked to neurologic disorders associated with dementia syndromes, such as Alzheimer's disease, Pick's disease, frontotemporal dementia, agryophillic grain disease, and progressive supranuclear palsy [56]. A recent genome-wide association study identified this gene as a risk factor for the development of familial PD [19]. Interestingly, ST13 and MAPT are downregulated in the female PD population indicating that these genes might also be implicated in a gender-specific development and progression of PD.

### Gene Expression Profiling in Postmortem Laser-Isolated Neurons

The interpretation of data from gene expression profiles based on microarrays or qRT-PCR on laser-isolated neurons from postmortem brain material bears several caveats. These include quality of postmortem brain tissue, sample preparation, methodology of laser-capture, quality of isolated and processed RNA, choice of microarray chip, probe design (e.g. detection of splice forms), array methodology, choice of primers, internal controls and method of quantitative Real-Time PCR, methodology of computational analysis including choice of algorithm, batch effects, cut-offs, replicates, and significance. Altogether, the complex interplay of these parameters represents a “dynamic” system in data generation and interpretation.

Restricted sample size is one of common problems in microarray studies of difficult-to-obtain sample populations, such as female sporadic PD in our study. Pathway enrichment can be used to compensate for this restriction, obtaining insightful information on the overall biological themes and overcoming common variations and fluctuations at the gene-level, which are embedded in data derived from either natural genetic variations or high-throughput experiments [14], [57]. We used two relevant methods that emphasize pathway-level enrichment patterns and consistency: Pathway pattern extraction pipeline (PPEP) and Sample-Level Enrichment-Based Pathway Ranking (SLEPR) [14], [57]. Both methods promote pathway-level comparative analysis of multiple gene lists or datasets on top of commonly used pathway enrichment analysis of a single gene list. Especially PPEP has been applied to a wide variety of different data sets, such as microarray data, mass spectrometry data, and data of genetic screening to uncover biological themes [58], [59], [60], [61], [62], [63], [64], [65], [66]. In our study, these analyses robustically revealed a male bias for signaling pathways relevant to PD pathogenesis, which otherwise would have been embedded amongst the variations at the gene-level, and demonstrated strong pathway enrichment in mPD using both stringent and relaxed analysis criteria, while females showed some enrichment at low levels only in the relaxed conditions. However, we also noticed that a gender effect was lost for some individual genes, such as PARK, when the relaxed criteria were applied - also attesting to the power of pathway-level comparative analysis. This demonstrated that the choice of cut-offs influenced gender-specificity on the single gene level that may be sensitive to genetic variations of individuals even from the same class (i.e. PD or normal control), but did not alter the overall outcome of the data set at large, which was retrieved by pathway-level comparative analysis. It should be noted that many of the excluded genes in the stringent criteria were “borderline cases” due to the narrow margin of significance applied by the cut-off shift from p<0.01 to p<0.05. Also, in case of genes, for which multiple probes were present, the two different stringencies selected different probes, from which the average values were calculated. For example, in mPD only two out of four probes for SNCA passed the p<0.05 cut-off, one being 2.1 fold down- and the other 1.3 fold up-regulated revealing a value of -1.13, further demonstrating the influence of multiple factors in data generation and gene-level assessment.

Conventional microarray analyses have contributed to the mechanistic understanding of complex disorders by attempting to determine conserved individual gene level changes. Pathway analysis has emerged as an alternative or compensation for conventional gene analysis, because it can cover both conserved and not conserved changes within small sample populations and can uncover changes that otherwise would have been overlooked by conventional methods. For example, conserved gene level changes may only exist in a subset of a sample population characterized by parameters such as race, aging, and others, but not in the variable of interest (i.e. gender in our analysis). Focusing on the “well behaved” or conserved gene changes alone would not only impede the choice of sample collection, but would also make conclusions and findings more subjective by selecting only those parameters that define the subsets of a population containing these conserved changes. In addition, it is common that relevant changes within certain phenotypes (in our study the differences in PD pathogenesis between genders) could occur for different genes in a relevant pathway across the individuals of a study population. Even for the same gene, changes could occur at different expression levels or aspects of function within different individuals, i.e., at the level of transcription, translation, or post-translational modification. Microarrays are just measuring one aspect of these many possible aspects. Since conserved gene-level changes are rather rare events, differ between individuals, and methodologically difficult to assess, pathway-level analysis detects multiple relevant gene changes in different individuals within the same phenotype (here, PD), which consists of the entire repertoire of all responsible changes eventually leading to the same phenotype: PD.

Conceptually, it is well accepted that biological processes are not based on single genes, but are rather a consequence of complex networks of signaling pathways that are driven by multiple gene functions. This is also true for disease processes and in particular in complex diseases, such as cancer, heart diseases, hypertension, and sporadic neurodegeneration, which have been associated with polymorphisms of multiple genes in the same or related pathway or caused by a single but different gene within individuals leading to biological changes in the same or related pathway among a given population. Pathway analysis offers the opportunity to deal with a group of relevant genes in such pathways instead of focusing on individual (conserved) gene level changes and, thus, could better reflect biological processes [58], [59], [60], [61], [62], [63], [64], [65], [66]. In this sense, data from pathway analysis could have implication on future aspects of understanding disease processes and could help to develop methodologies for assessing the outcome of pathway damage from sample to sample eventually determining which set of genes could be candidate targets to be screened and identified for diagnosis, treatment and even disease prevention.

Data validation using qRT-PCR demonstrated an overall good correlation of array raw data (normalized values in z-scores) with the qRT-PCR results (2^−ΔCt^ method [16]) across all samples. However, despite this data confirmation, the validation on a small number of genes and samples was less evident when compared with the array data after analysis with SAM. Although there was a trend for some genes towards stronger down-regulation in males, overall the PCR revealed similar reduction of gene expression between control and PD samples for both genders. Interestingly, these results correlated better with the relaxed p<0.05 criteria and, therefore, point to a general caveat in computational data analysis of determining appropriate cut-offs. Despite the results from the pathway enrichment analysis, the validation of potentially conserved individual gene level changes by qRT-PCR would require further studies on additional biological replicates including a larger amount of female subjects. Finally, the PCR experiments also confirmed our previous observation that gene expression levels substantially varied between individual genes as well as subjects and some of the genes, such as TH, Parkin, LRKK2, RIMS1 and 3 were very low expressed. This could be an important parameter to put into perspective some of the array results and especially for those genes that are expressed at very low levels.

We attempted to re-analyze the microarray data published by Cantuti-Castelvetri et al. [11] according to our criteria. These investigators also analyzed the gene expression profiles of laser captured DA neurons from female and male normal subjects and sporadic PD patients and reported dissimilarity of gene deregulation patterns in signal transduction, neuronal maturation, protein kinases, proteolysis and the WNT signaling pathway with a predisposition of males to PD. Unfortunately, using our stringent and relaxed analysis conditions we were not able to retrieve a comprehensive data set that allowed for a comparative analysis with our results: For example, the group of mN_fN contained 305 genes with 301 upregulated probesets. In female PD (fN_fPD) only 5 probesets were present in both the p<0.01 and p<0.05 conditions, while in mPD (mN_mPD) 175 probesets were found with p-values <0.01 (172 were down-regulated) and 1241 with p<0.05. When we looked at the deregulated genes in these groups, the majority was not relevant to PD signaling pathways. These discrepancies could have been due to some substantial differences between both studies: 1.) As already discussed previously [12], Cantuti-Castelvetri et al. used laser-capture microscopy (LCM) with an Arcturus PixCell II instrument and a different sample preparation protocol based on quick immunostaining or ethanol fixation and methylene blue staining. In addition, this study was based on the Affymetrix U133_X3P platform, which consists of a different set of probesets than the HG-U133A chip used in our study. 2.) Our computational analysis takes in account two important parameters for data transformation: Batch effect and Principal Component Analysis (PCA). Consistent with our ANOVA analysis [12], PCA plots of data after removal of a batch effect by an ANOVA model showed a clear pattern formed by the PD versus normal samples. This, however, was not the case for the data by Cantuti-Castelvetri et al. and indicated profound differences in the sets of microarray samples between both studies. It should be noted that, on the single gene level, dissimilarities between the two microarrays could also be observed in an independent computational analysis publicized on the National Center for Adult Stem Cell Research (NCASCR) - Parkinson's Disease Review Database (https://ncascr.griffith.edu.au/pdreview/2009/).

Taken together, our data provide insight into some of the molecular events of sporadic PD and a potential role of gender in disease pathogenesis. The results could support the notion that the male gender is a risk factor for PD and could indicate a profound impact of gender on the function of normal and PD-affected DA neurons. Moreover, they imply that a relatively small set of primary factors could represent key molecules that drive disease mechanisms and gender-specificity. Altogether, we believe that our data provide a platform for future investigations on understanding the role of gender in PD pathogenesis.

## Supporting Information

Table S1 Statistics of cases used for LMD and RNA information.
(0.11 MB DOC)

Table S2 (2.14 MB XLS)

Table S3 (3.40 MB XLS)

Table S4 (0.13 MB XLS)

Table S5 (0.54 MB XLS)

Figure S1 Heatmaps based on FDR5 p<0.01 of all 6 gene lists (Table S2) merged by BaseGenBankID of WPS. In case of multiple probes for the same gene, data are plotted as the average of fold change for each gene.
(0.04 MB PPT)

Figure S2 Distribution of gene expression profiles for all groups based on FDR5 p<0.05 analysis as shown in Figure 1. The groups are as follows: allN_allPD compares all normal versus all PD samples; mN_fN compares control males versus control females; mN_mPD compares control males versus male PD; fN_fPD compares control females versus female PD; the overlapping groups indicate the number of genes that are found in several respective groups. Bars represent numbers of up- or downregulated genes in each group for total genes or after setting the cut-off of differential gene expression at >1.5 fold.
(0.18 MB PPT)

Figure S3 Comparative pathway-enrichment level analysis based on FDR5 p<0.01 using BioCarta (A) and KEGG (B) for all gene lists (Table S2). There was more enrichment of probesets in pathways relevant to PD pathogenesis in the mPD gene list (mN_mPD).
(0.09 MB PPT)

Figure S4 Comparative pathway-enrichment level analysis based on FDR5 p<0.01 using GO-BP for all lists (A), allN_allPD with mN_mPD (B), and allN_allPD with fN_fPD (C) (Table S2). There was prominent enrichment of probesets in pathways relevant to PD pathogenesis in mPD. Note that allN_allPD A3W depicts probesets from the ANOVA FDR10 gene list previously published [12].
(0.24 MB PPT)

Figure S5 Comparative pathway-enrichment level analysis based on FDR5 p<0.01 using GSEA function annotation for all lists (A), allN_allPD with mN_mPD (B), and allN_allPD with fN_fPD (C) (Table S2). There was prominent enrichment of probesets in pathways relevant to PD pathogenesis in mPD. Note that allN_allPD A3W depicts probesets from the ANOVA FDR10 gene list previously published [12].
(0.30 MB PPT)

Figure S6 Comparative pathway-enrichment level analysis based on FDR5 p<0.01 and p<0.05 for all gene lists. (A) The complete list of terms for GO-BP and GSEA showed more consistent enrichment for allN_allPD and mN_mPD than for fN_fPD. (B–D) Selected lists for GO-BP (B), GSEA (C) and KEGG (D) analysis demonstrated stronger and more prominent enrichment of terms (arrows) related to oxidative phosphorylation, synaptic transmission and transmission of nerve impulse in mPD than in fPD, but to lesser extend for apoptosis.
(1.16 MB PPT)

Figure S7 Comparison of gene expression levels in the microarrays before data transformation (normalized values in z-scores) with data from the qRT-PCR assays. (A–B) Heat maps of z-scores for the 14 genes analyzed in all individual samples after removal of batch effects (Material and Methods and [12]). The heatmaps in (B) depict the differences from mean levels. (C–E) Scatter plots summarizing the values for the z-scores in comparison with the values of relative gene expression (2^−ΔCt^) from the qRT-PCR results. Black dots represent control and red dots PD samples. Note, that genes were clustered according to high (DAT, UCHL1), medium (Girk2, SNCA, HIP2, DJ-1, ATP13A2, RAP1GA1), and low (TH, Parkin, PINK1, LRRK2, RIMS1 and 3) expressing genes.
(0.37 MB PPT)
