# Supplementary material for: Evidence for Gender-Specific Transcriptional Profiles of Nigral Dopamine Neurons in Parkinson Disease
Source: PLoS One. 2010 Jan 25;5(1):e8856. doi: 10.1371/journal.pone.0008856 (PMC2810324; doi:10.1371/journal.pone.0008856)
Supplement: Table S1 — Statistics of cases used for LMD and RNA information. (0.11 MB DOC) [file pone.0008856.s001.doc]

Table S1:

**Statistics of cases used for LMD and RNA information**

| **Case ID** | **Assay ID^1^** | **Age** | **Primary Diagnosis** | **Gender** | PMI | RatioGAPDH^1^ | Ratioβ-actin^1^ | Ratio **RNA^1^** | % probe **sets^1^** | **Ratio**  **260/280^2^** | Assay |
| --- | --- | --- | --- | --- | --- | --- | --- | --- | --- | --- | --- |
| C1 | 1020 | 73 | Control | M | 20.53 | 33.0 | 112.0 | 1.46 | 19.2 | 1.67 | array/PCR |
| C2 | 1022 | 89 | Control | M | 7.4 | 1.09 | 3.64 | 1.0 | 7.7 | 2.47 | array/PCR |
| C3 | 1024 | 79 | Control | M | 20.92 | 2.35 | 6.51 | 0.6 | 4.9 | 1.69 | array/PCR |
| C4 | 1147 | 78 | Control | M | 21.75 | 3.53 | 4.42 | 0.98 | 10.7 | 1.73 | array/PCR |
| C5 | 1150 | 75 | Control | M | 20.12 | 2.38 | 11.3 | 1.33 | 8.6 |  | array |
| C6 | 1151 | 68 | Control | M | 16.58 | 1.18 | 2.94 | 1.24 | 8.3 |  | array |
| C7 | 1152 | 72 | Control | F | 18.25 | 1.76 | 1.12 | 0.45 | 2.7 |  | array |
| C8 | 1156 | 69 | Control | F | 25.15 | 6.84 | 8.71 | 1.04 | 11.9 | 1.69 | array/PCR |
| C9 | 1157 | 74 | Control | F | 12.17 | 15.47 | 22.22 | 0.83 | 21.6 | 1.73 | array/PCR |
| C10 |  | 72 | Control | M | 18.25 |  |  |  |  | 1.73 | PCR |
| C11 |  | 71 | Control | M | 23.40 |  |  |  |  | 1.75 | PCR |
| PD1 | 1143 | 77 | PD | M | 10.33 | 1.2 | 1.71 | 0.46 | 4.3 | 1.2 | array/PCR |
| PD2 | 1144 | 81 | PD | F | 17 | 0.33 | 2.64 | 1.14 | 3.4 |  | array |
| PD3 | 1145 | 79 | PD | M | 23.42 | 2.28 | 1.59 | 0.52 | 5.7 | 1.73 | array/PCR |
| PD4 | 1146 | 72 | PD | M | 26.25 | 2.29 | 16.35 | 0.96 | 7.7 | 1.78 | array/PCR |
| PD5 | 1148 | 73 | PD | M | 18 | 0.33 | 1.3 | 0.46 | 4.1 |  | array |
| PD6 | 1149 | 83 | PD | M | 21.25 | 1.71 | 6.36 | 0.89 | 6.0 | 2.79 | array/PCR |
| PD7 | 1153 | 77 | PD | M | 22.67 | 1.97 | 2.33 | 0.78 | 3.4 |  | array |
| PD8 | 1154 | 84 | PD | F | 6.42 | 6.05 | 4.03 | 0.92 | 7.1 | 1.82 | array/PCR |
| PD9 | 1155 | 77 | PD | M | 26.25 | 1.49 | 2.36 | 0.77 | 6.1 |  | array |
| PD10 | 1158 | 81 | PD | F | 26.75 | 2.03 | 4.62 | 0.15 | 5.6 | 4.88 | array/PCR |
| PD11 |  | 68 | PD | M | 13.92 |  |  |  |  | 1.73 | PCR |
| PD13 |  | 81 | PD | F | 22.75 |  |  |  |  | 1.44 | PCR |
| PD14 |  | 77 | PD | M | 28.16 |  |  |  |  | 1.55 | PCR |
| **Group** | **Average**  **Age^3^** | | **Average**  **PMI^3^** |  | | | | | | | |
| fC | 71.6 | | 18.5 |  | | | | | | | |
| mC | 75.6 | | 18.6 |  | | | | | | | |
| fPD | 81.7 | | 18.2 |  | | | | | | | |
| mPD | 75.9 | | 21.1 |  | | | | | | | |

**^1^**mRNA array ID and RNA information derived from: <http://national_databank.mclean.harvard.edu/brainbank>

**^2^**260/280 ratios determined by nanodrop OD measurements after RNA purification from LMD cells

**^3^**average from all female or male control or PD cases
