# Supplementary figures and images for: Evidence for Gender-Specific Transcriptional Profiles of Nigral Dopamine Neurons in Parkinson Disease
Source: PLoS One. 2010 Jan 25;5(1):e8856. doi: 10.1371/journal.pone.0008856 (PMC2810324; doi:10.1371/journal.pone.0008856)

## Slide 1
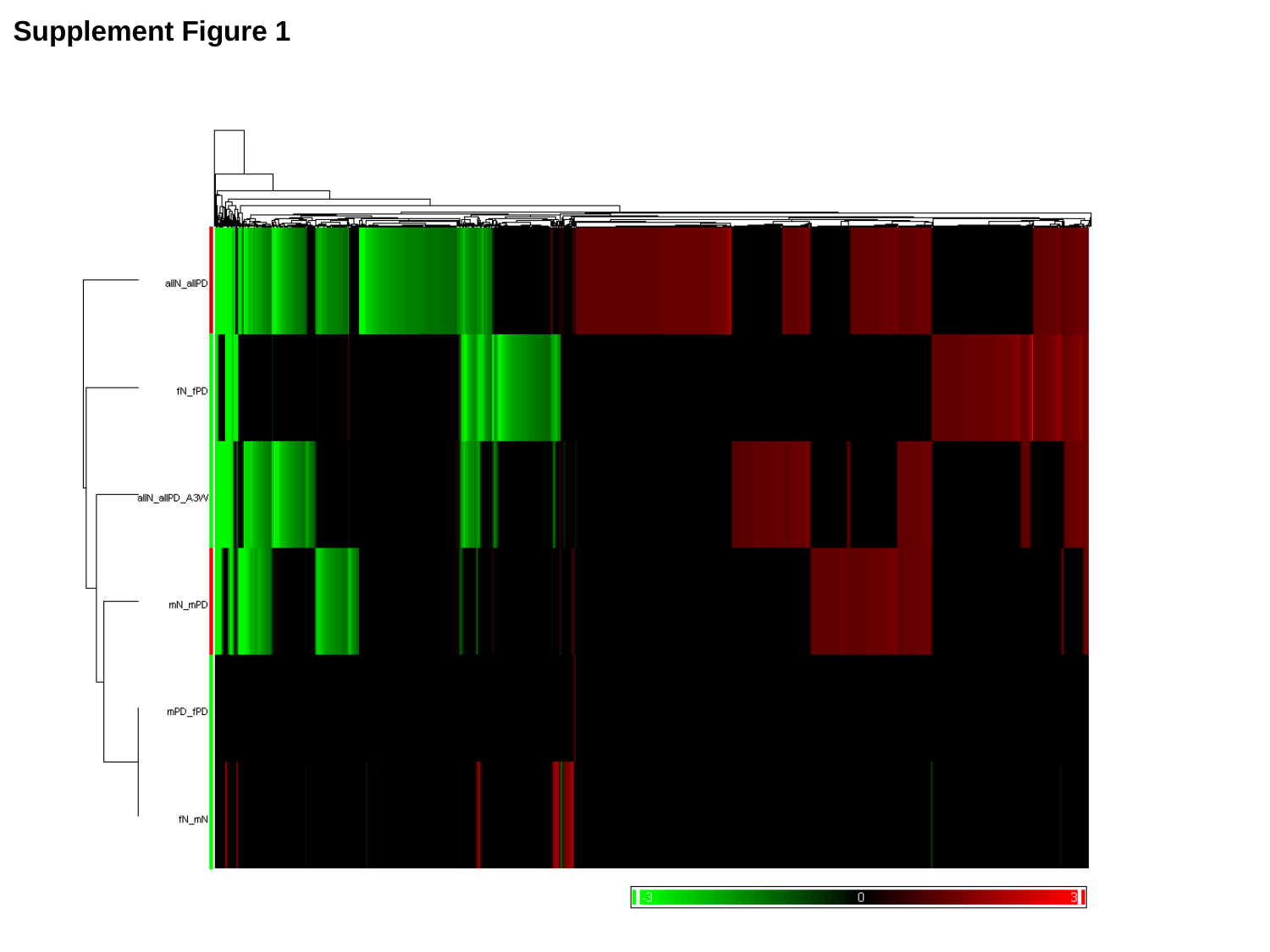

Supplement Figure 1

Supplement: Figure S1 — Heatmaps based on FDR5 p<0.01 of all 6 gene lists (Table S2) merged by BaseGenBankID of WPS. In case of multiple probes for the same gene, data are plotted as the average of fold change for each gene. (0.04 MB PPT) [file pone.0008856.s006.ppt]
