# Supplementary material for: Evidence for Gender-Specific Transcriptional Profiles of Nigral Dopamine Neurons in Parkinson Disease
Source: PLoS One. 2010 Jan 25;5(1):e8856. doi: 10.1371/journal.pone.0008856 (PMC2810324; doi:10.1371/journal.pone.0008856)
Supplement: Figure S2 — Distribution of gene expression profiles for all groups based on FDR5 p<0.05 analysis as shown in Figure 1. The groups are as follows: allN_allPD compares all normal versus all PD samples; mN_fN compares control males versus control females; mN_mPD compares control males versus male PD; fN_fPD compares control females versus female PD; the overlapping groups indicate the number of genes that are found in several respective groups. Bars represent numbers of up- or downregulated genes in each group for total genes or after setting the cut-off of differential gene expression at >1.5 fold. (0.18 MB PPT) [file pone.0008856.s007.ppt]

## Slide 1
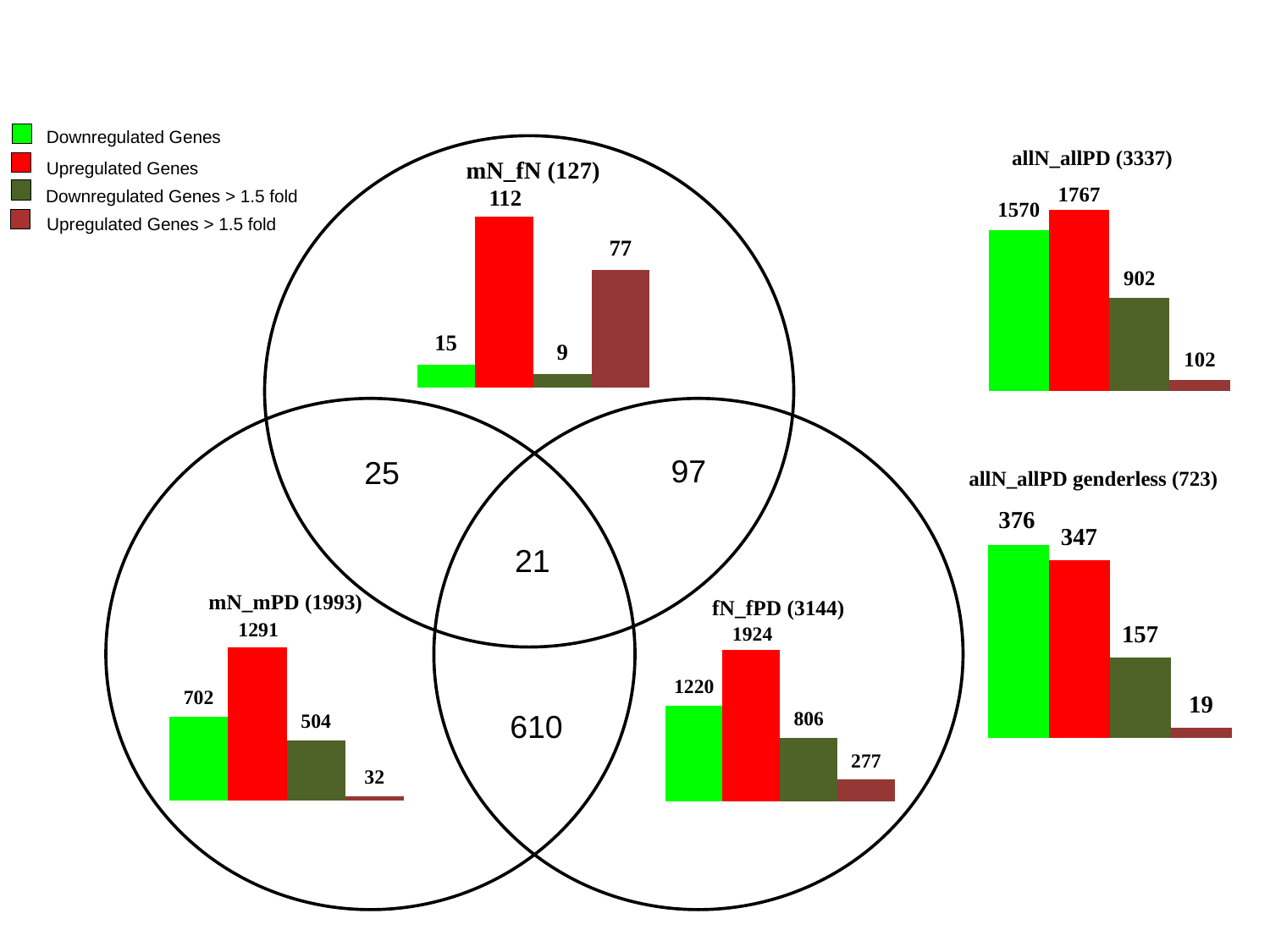

Downregulated Genes
Upregulated Genes
Downregulated Genes > 1.5 fold
Upregulated Genes > 1.5 fold
allN_allPD (3337)
97
25
allN_allPD genderless (723)
21
610
