# Supplementary material for: Evidence for Gender-Specific Transcriptional Profiles of Nigral Dopamine Neurons in Parkinson Disease
Source: PLoS One. 2010 Jan 25;5(1):e8856. doi: 10.1371/journal.pone.0008856 (PMC2810324; doi:10.1371/journal.pone.0008856)
Supplement: Figure S3 — Comparative pathway-enrichment level analysis based on FDR5 p<0.01 using BioCarta (A) and KEGG (B) for all gene lists (Table S2). There was more enrichment of probesets in pathways relevant to PD pathogenesis in the mPD gene list (mN_mPD). (0.09 MB PPT) [file pone.0008856.s008.ppt]

## Slide 1
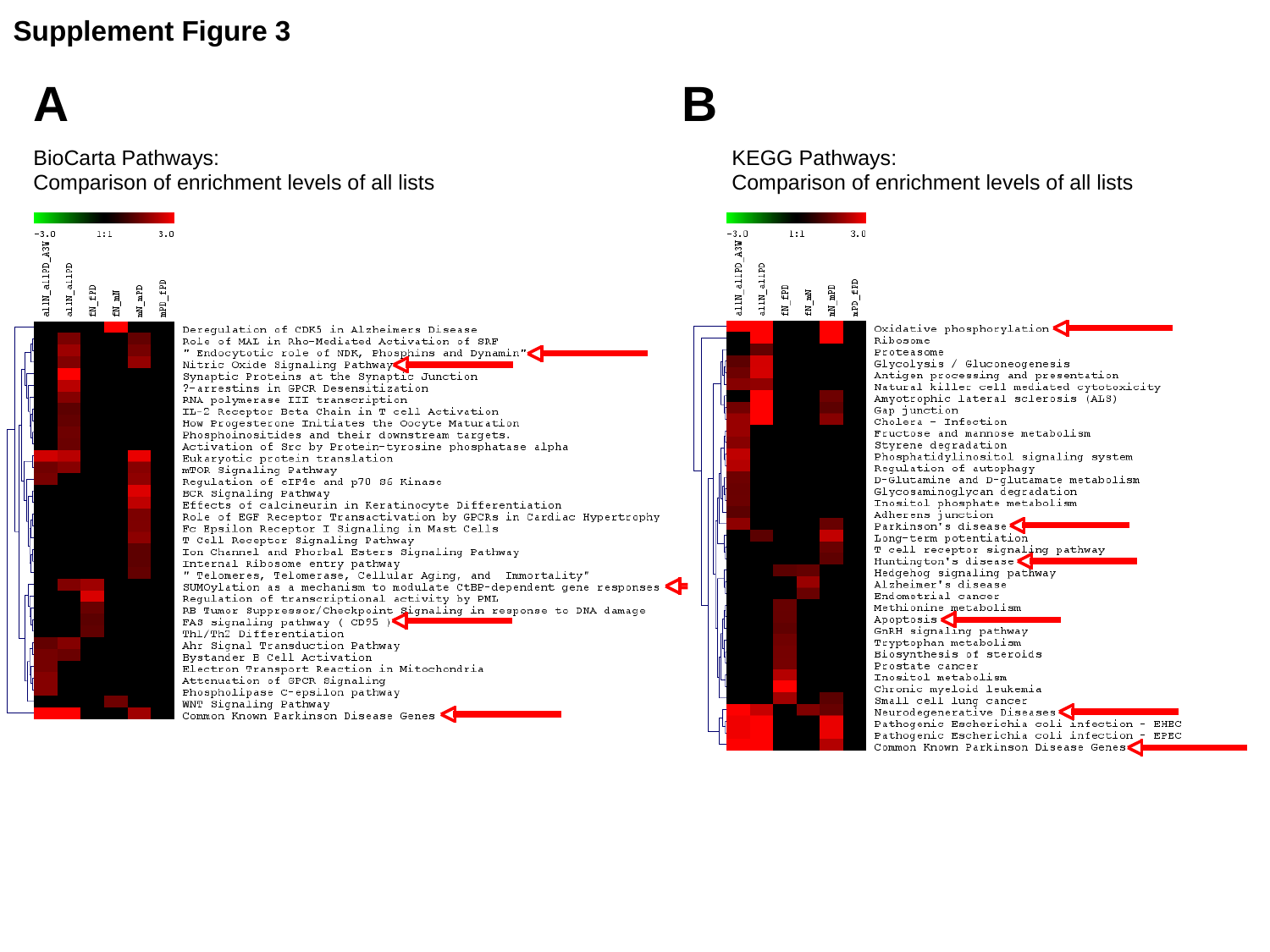

Supplement Figure 3
A
B
BioCarta Pathways:
Comparison of enrichment levels of all lists
KEGG Pathways:
Comparison of enrichment levels of all lists
