# Supplementary material for: Evidence for Gender-Specific Transcriptional Profiles of Nigral Dopamine Neurons in Parkinson Disease
Source: PLoS One. 2010 Jan 25;5(1):e8856. doi: 10.1371/journal.pone.0008856 (PMC2810324; doi:10.1371/journal.pone.0008856)
Supplement: Figure S4 — Comparative pathway-enrichment level analysis based on FDR5 p<0.01 using GO-BP for all lists (A), allN_allPD with mN_mPD (B), and allN_allPD with fN_fPD (C) (Table S2). There was prominent enrichment of probesets in pathways relevant to PD pathogenesis in mPD. Note that allN_allPD A3W depicts probesets from the ANOVA FDR10 gene list previously published [12]. (0.24 MB PPT) [file pone.0008856.s009.ppt]

## Slide 1
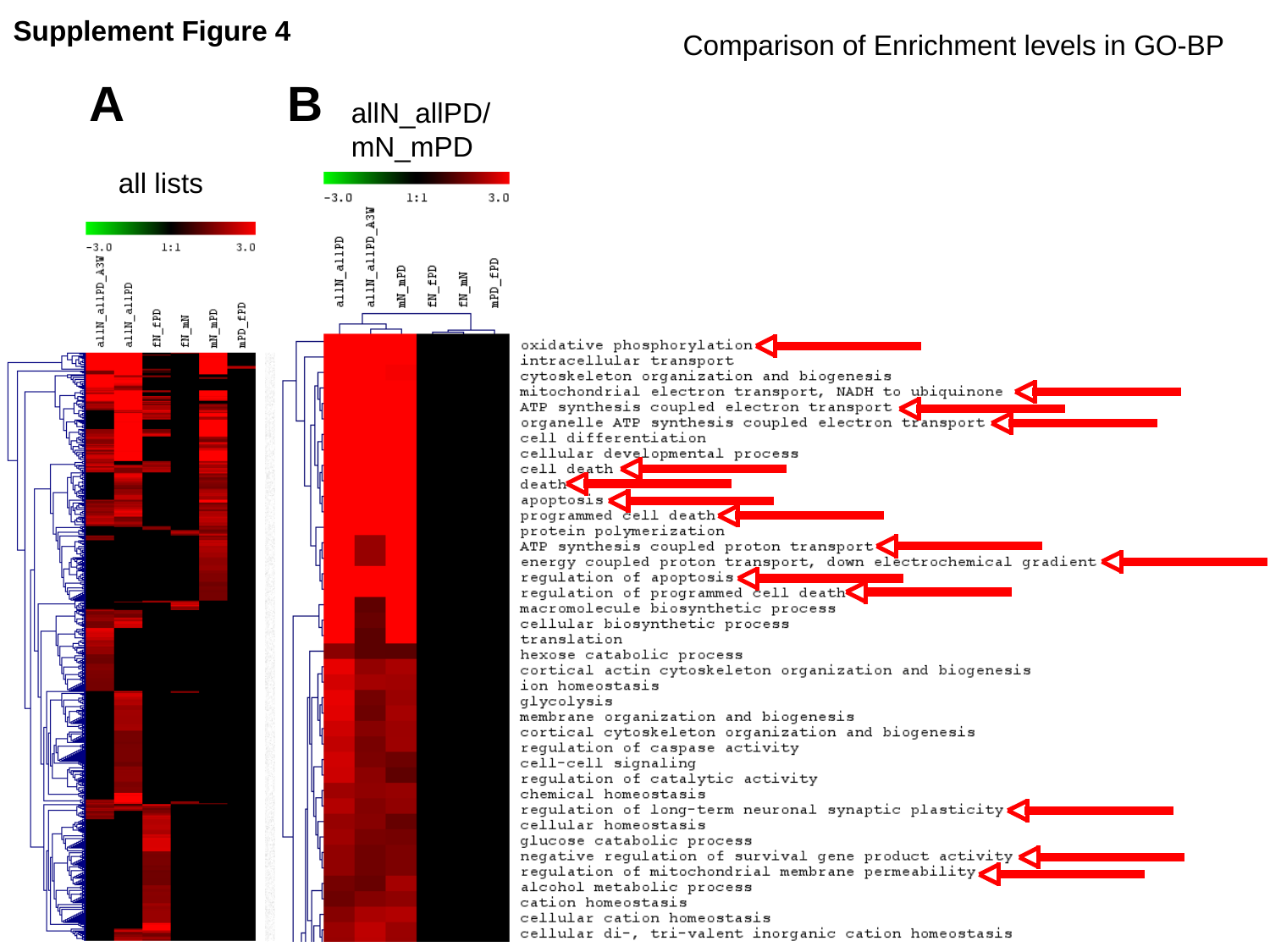

Supplement Figure 4
Comparison of Enrichment levels in GO-BP
A
B
allN_allPD/
mN_mPD
all lists

## Slide 2
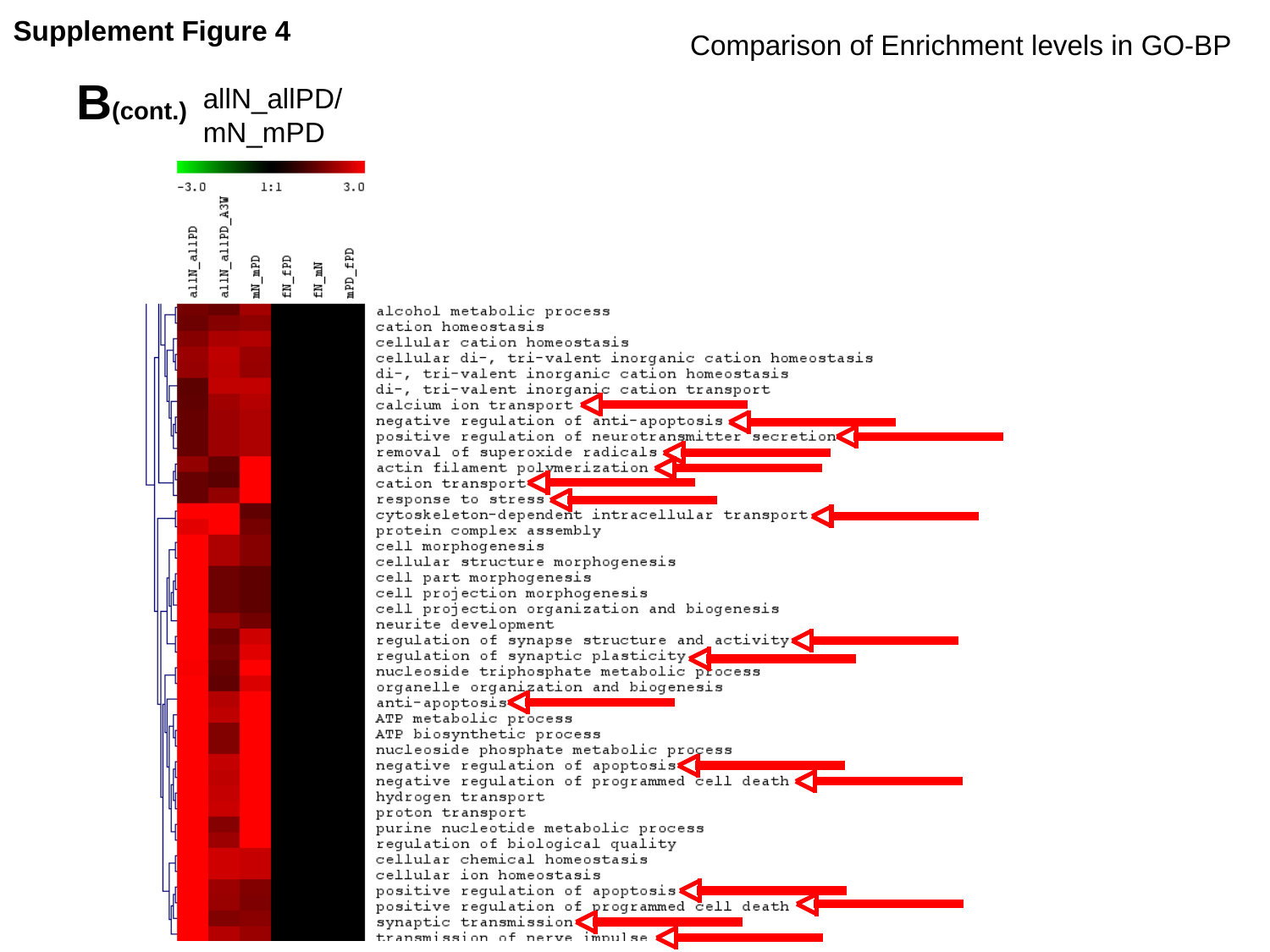

Supplement Figure 4
Comparison of Enrichment levels in GO-BP
B(cont.)
allN_allPD/
mN_mPD

## Slide 3
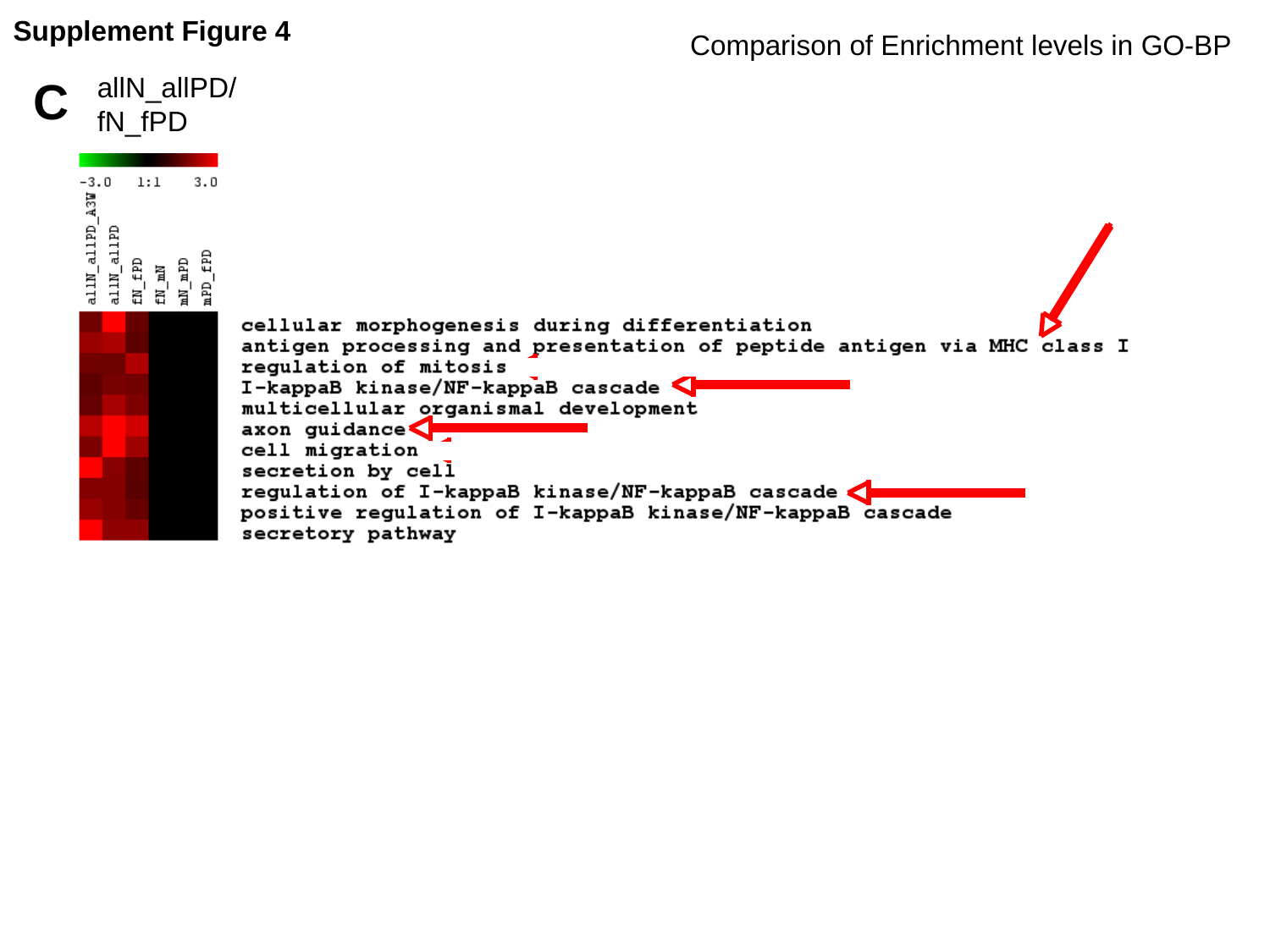

Supplement Figure 4
Comparison of Enrichment levels in GO-BP
C
allN_allPD/
fN_fPD
