# Supplementary material for: Evidence for Gender-Specific Transcriptional Profiles of Nigral Dopamine Neurons in Parkinson Disease
Source: PLoS One. 2010 Jan 25;5(1):e8856. doi: 10.1371/journal.pone.0008856 (PMC2810324; doi:10.1371/journal.pone.0008856)
Supplement: Figure S6 — Comparative pathway-enrichment level analysis based on FDR5 p<0.01 and p<0.05 for all gene lists. (A) The complete list of terms for GO-BP and GSEA showed more consistent enrichment for allN_allPD and mN_mPD than for fN_fPD. (B–D) Selected lists for GO-BP (B), GSEA (C) and KEGG (D) analysis demonstrated stronger and more prominent enrichment of terms (arrows) related to oxidative phosphorylation, synaptic transmission and transmission of nerve impulse in mPD than in fPD, but to lesser extend for apoptosis. (1.16 MB PPT) [file pone.0008856.s011.ppt]

## Slide 1
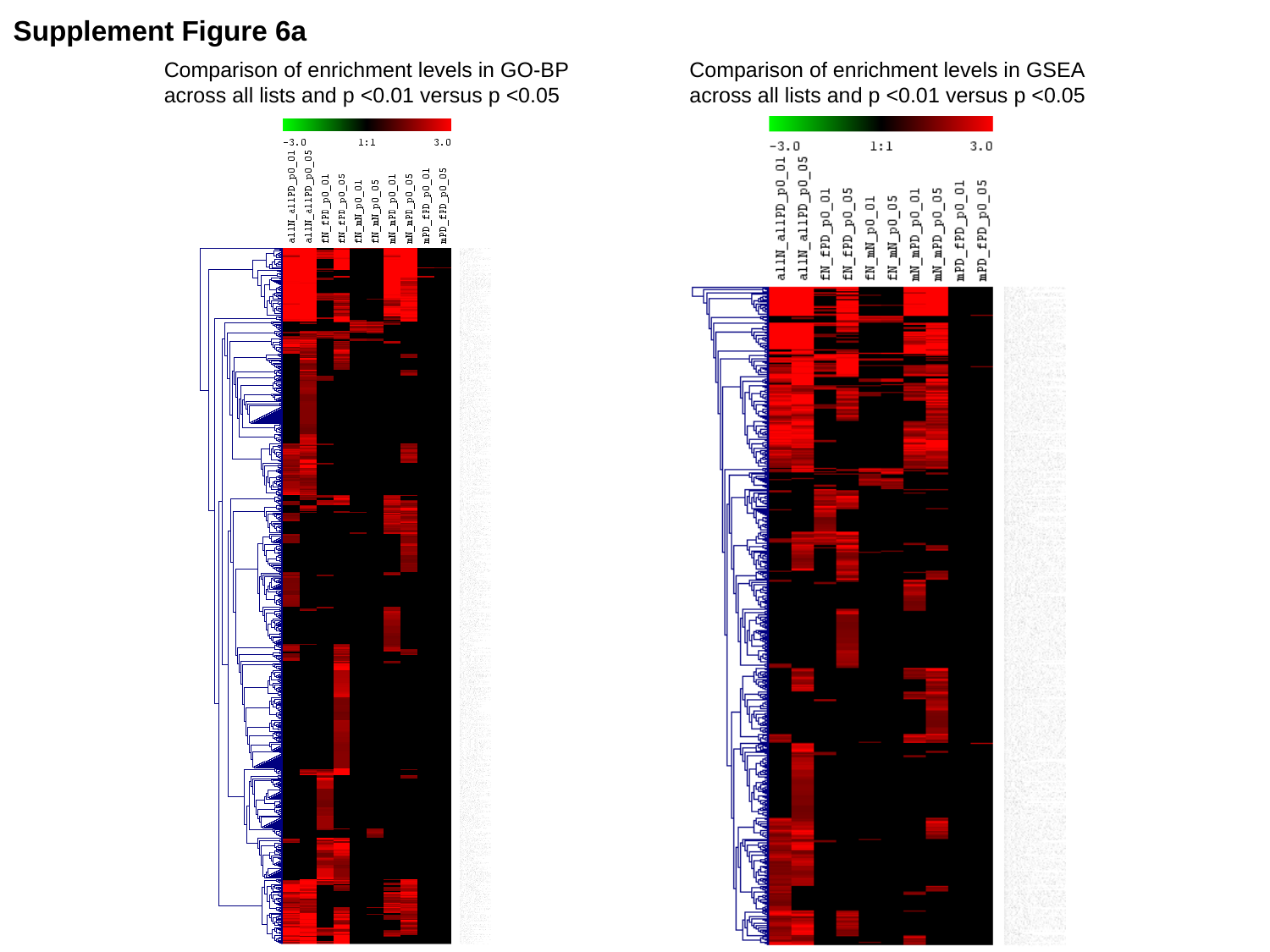

Supplement Figure 6a
Comparison of enrichment levels in GO-BP
across all lists and p <0.01 versus p <0.05
Comparison of enrichment levels in GSEA
across all lists and p <0.01 versus p <0.05

## Slide 2
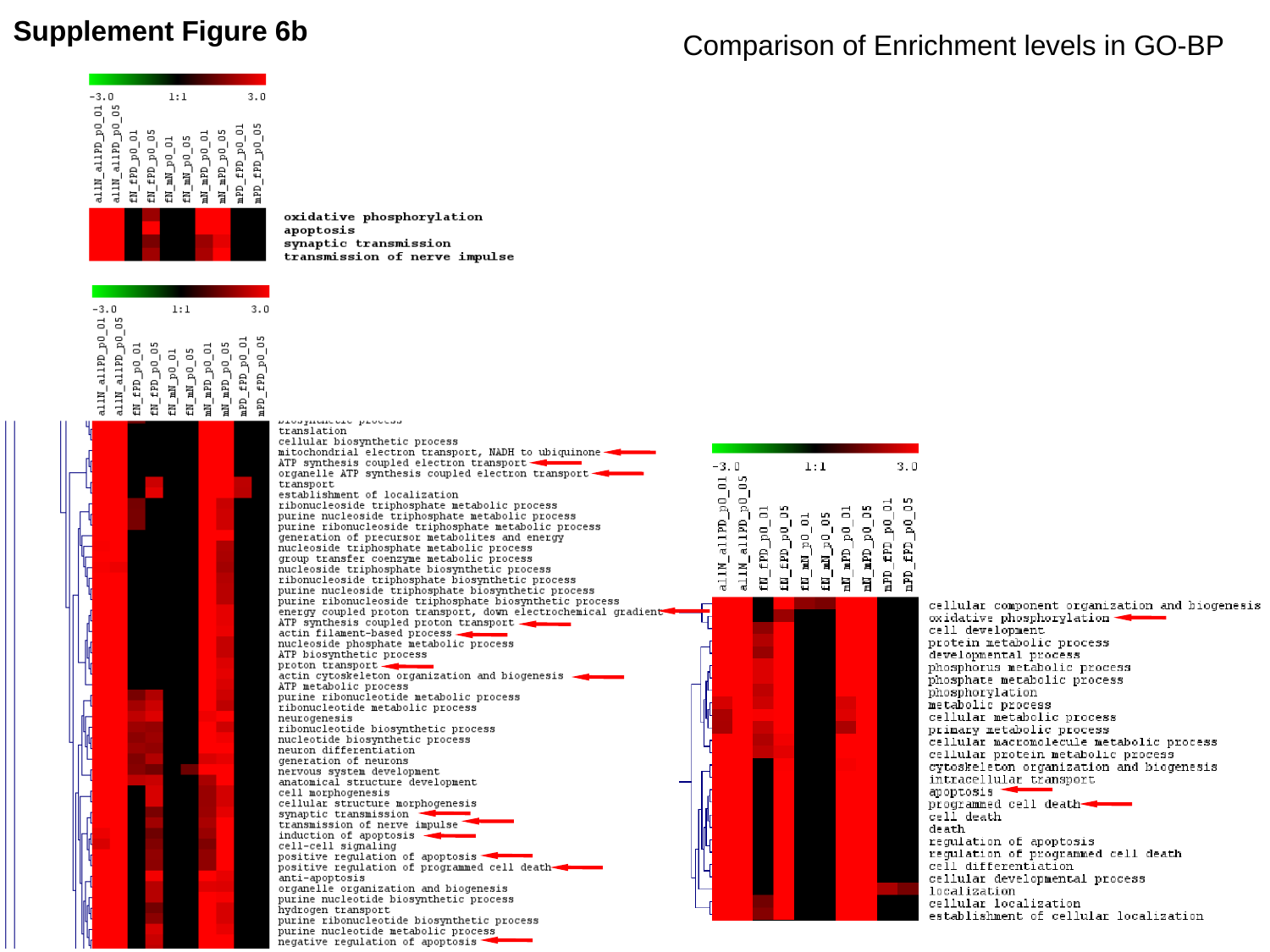

Supplement Figure 6b
Comparison of Enrichment levels in GO-BP

## Slide 3
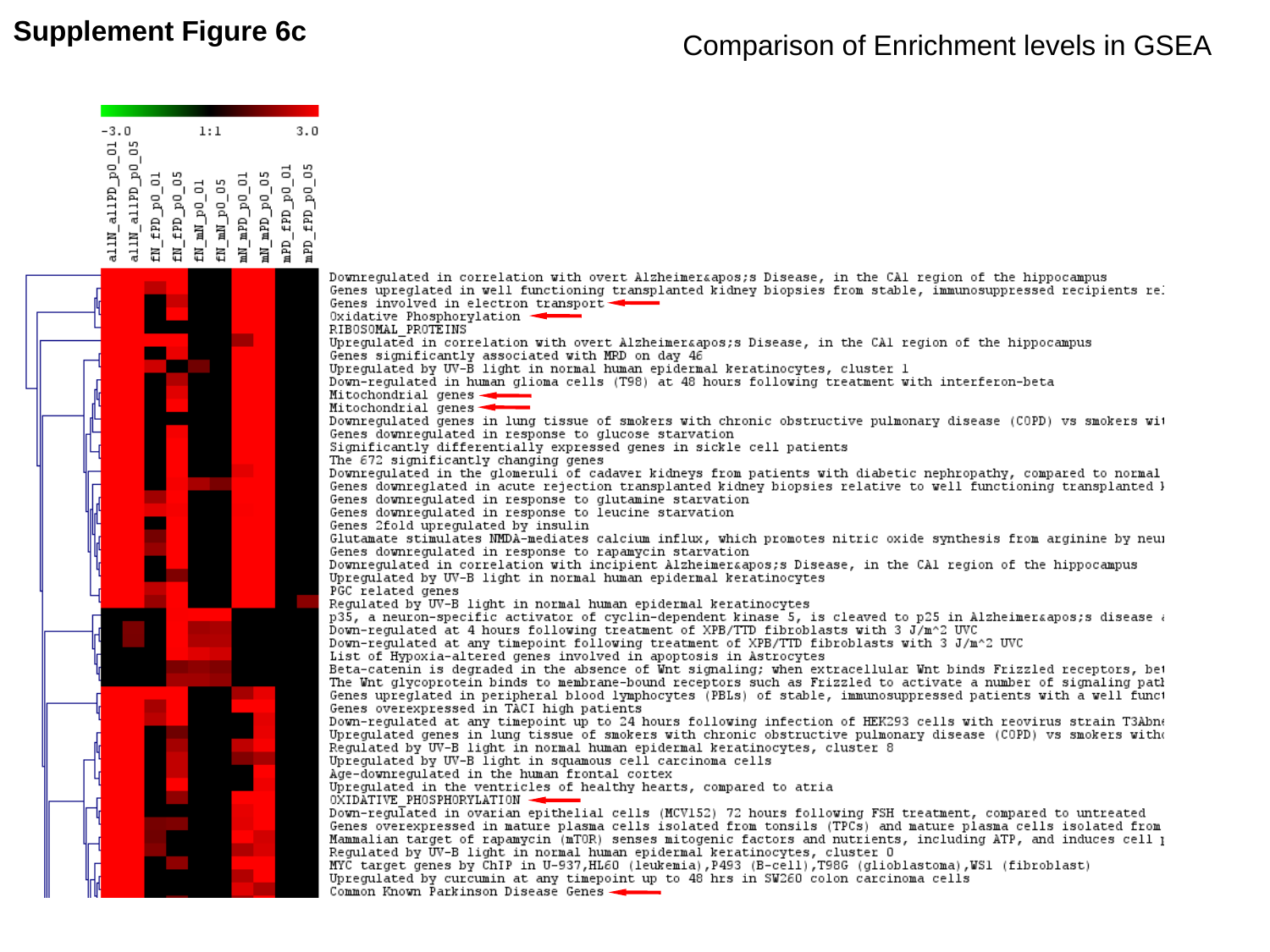

Supplement Figure 6c
Comparison of Enrichment levels in GSEA

## Slide 4
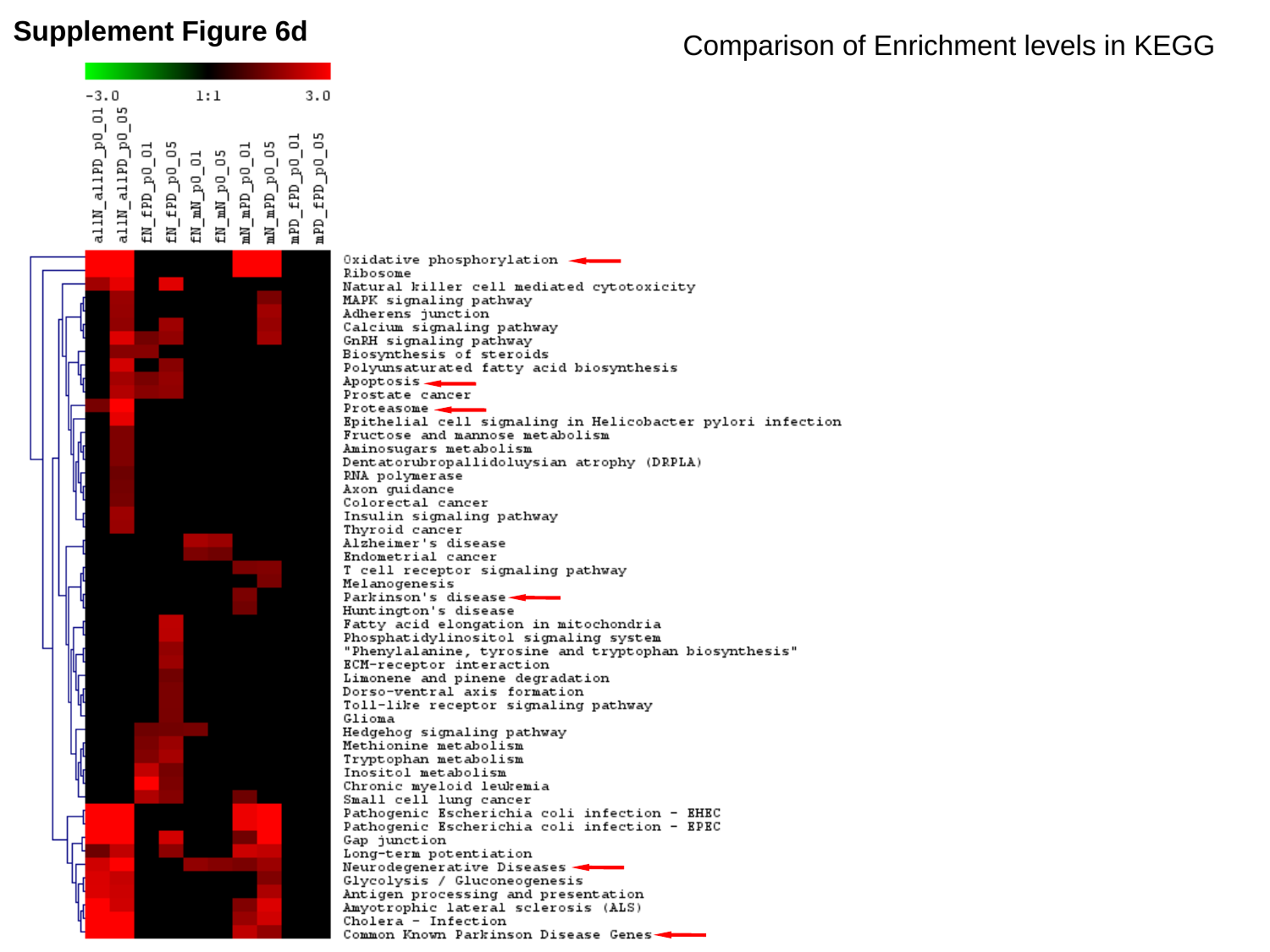

Supplement Figure 6d
Comparison of Enrichment levels in KEGG
