# Supplementary material for: Evidence for Gender-Specific Transcriptional Profiles of Nigral Dopamine Neurons in Parkinson Disease
Source: PLoS One. 2010 Jan 25;5(1):e8856. doi: 10.1371/journal.pone.0008856 (PMC2810324; doi:10.1371/journal.pone.0008856)
Supplement: Figure S7 — Comparison of gene expression levels in the microarrays before data transformation (normalized values in z-scores) with data from the qRT-PCR assays. (A–B) Heat maps of z-scores for the 14 genes analyzed in all individual samples after removal of batch effects (Material and Methods and [12]). The heatmaps in (B) depict the differences from mean levels. (C–E) Scatter plots summarizing the values for the z-scores in comparison with the values of relative gene expression (2−ΔCt) from the qRT-PCR results. Black dots represent control and red dots PD samples. Note, that genes were clustered according to high (DAT, UCHL1), medium (Girk2, SNCA, HIP2, DJ-1, ATP13A2, RAP1GA1), and low (TH, Parkin, PINK1, LRRK2, RIMS1 and 3) expressing genes. (0.37 MB PPT) [file pone.0008856.s012.ppt]

## Slide 1
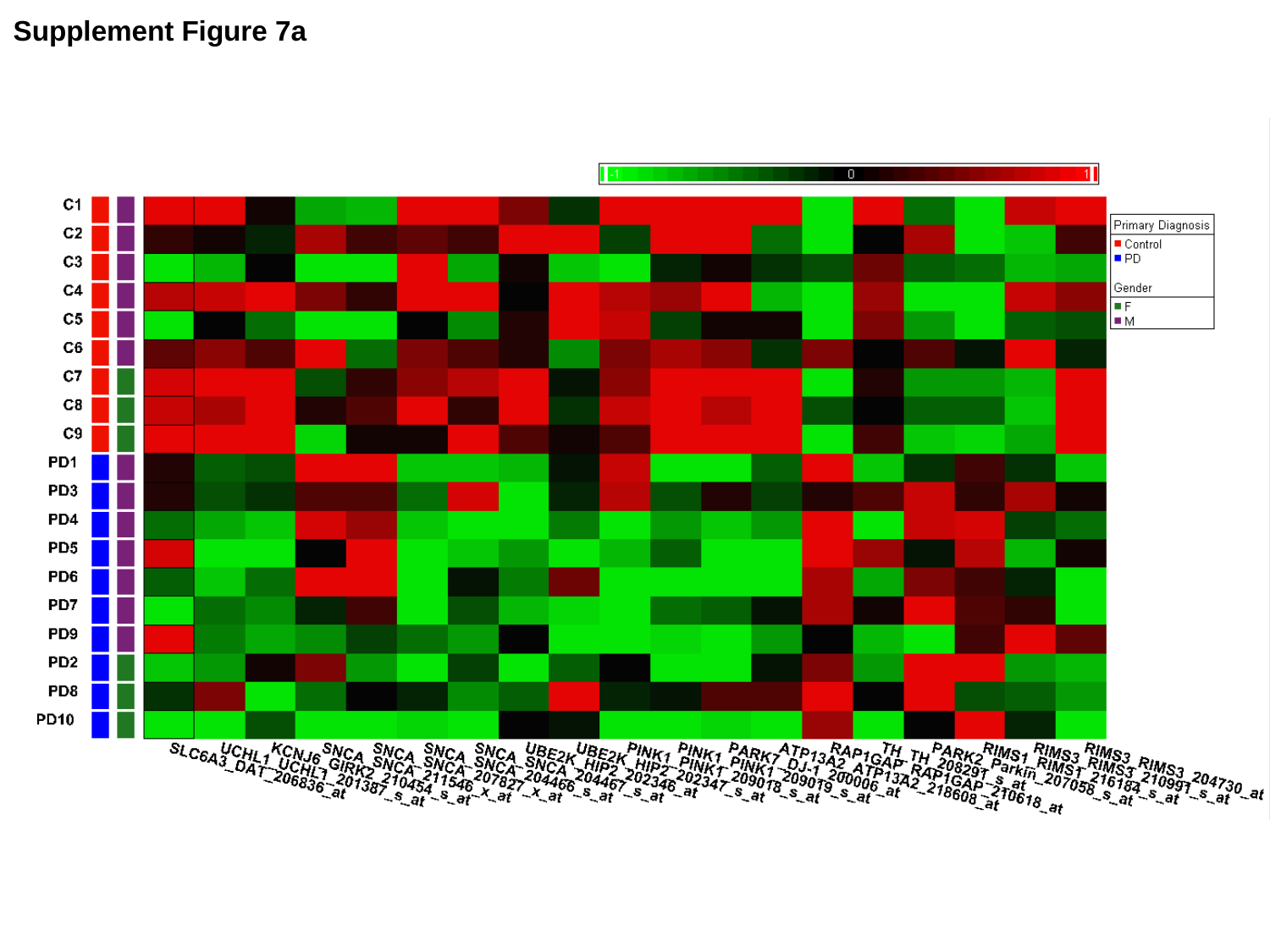

Supplement Figure 7a

## Slide 2
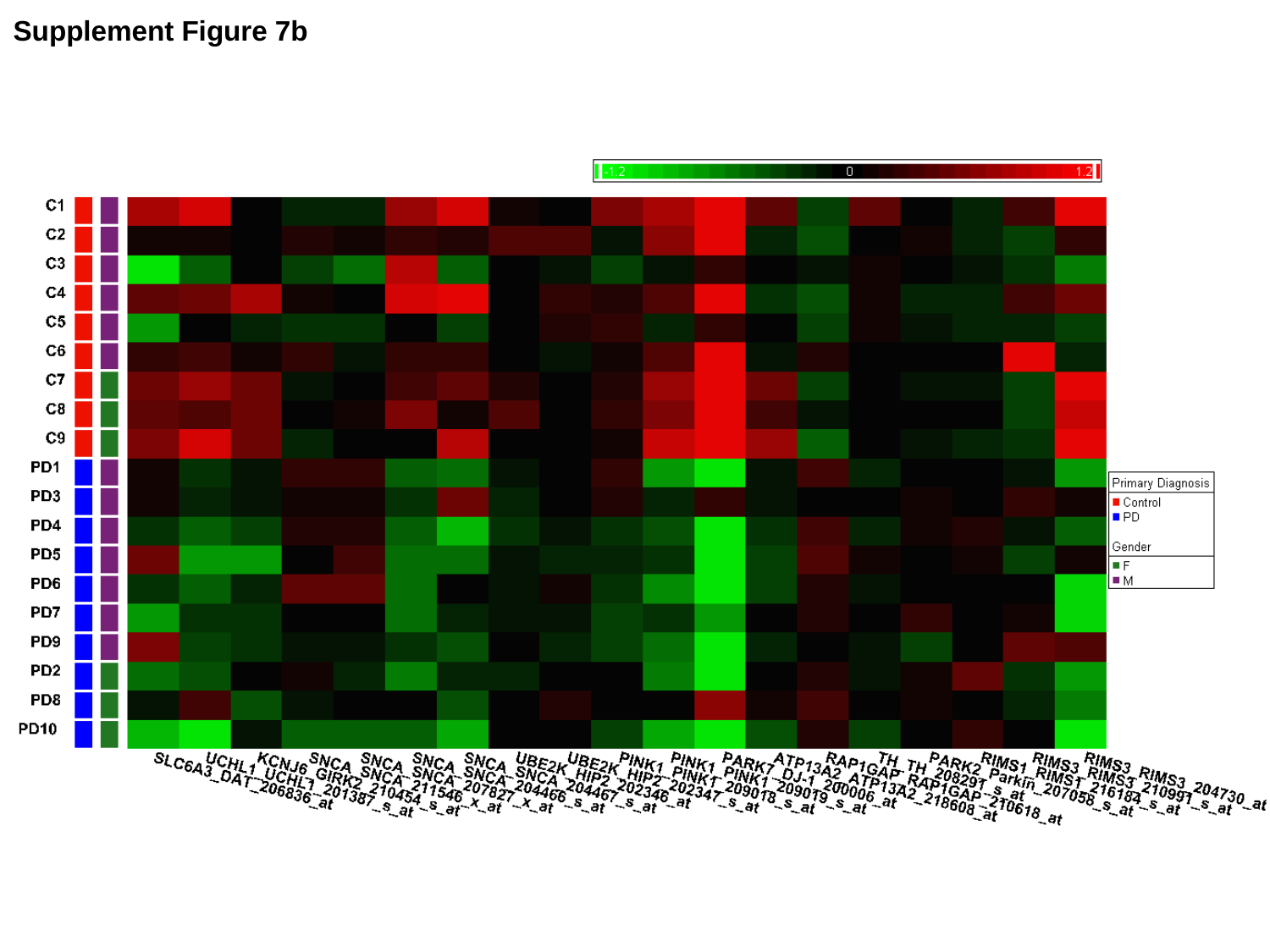

Supplement Figure 7b

## Slide 3
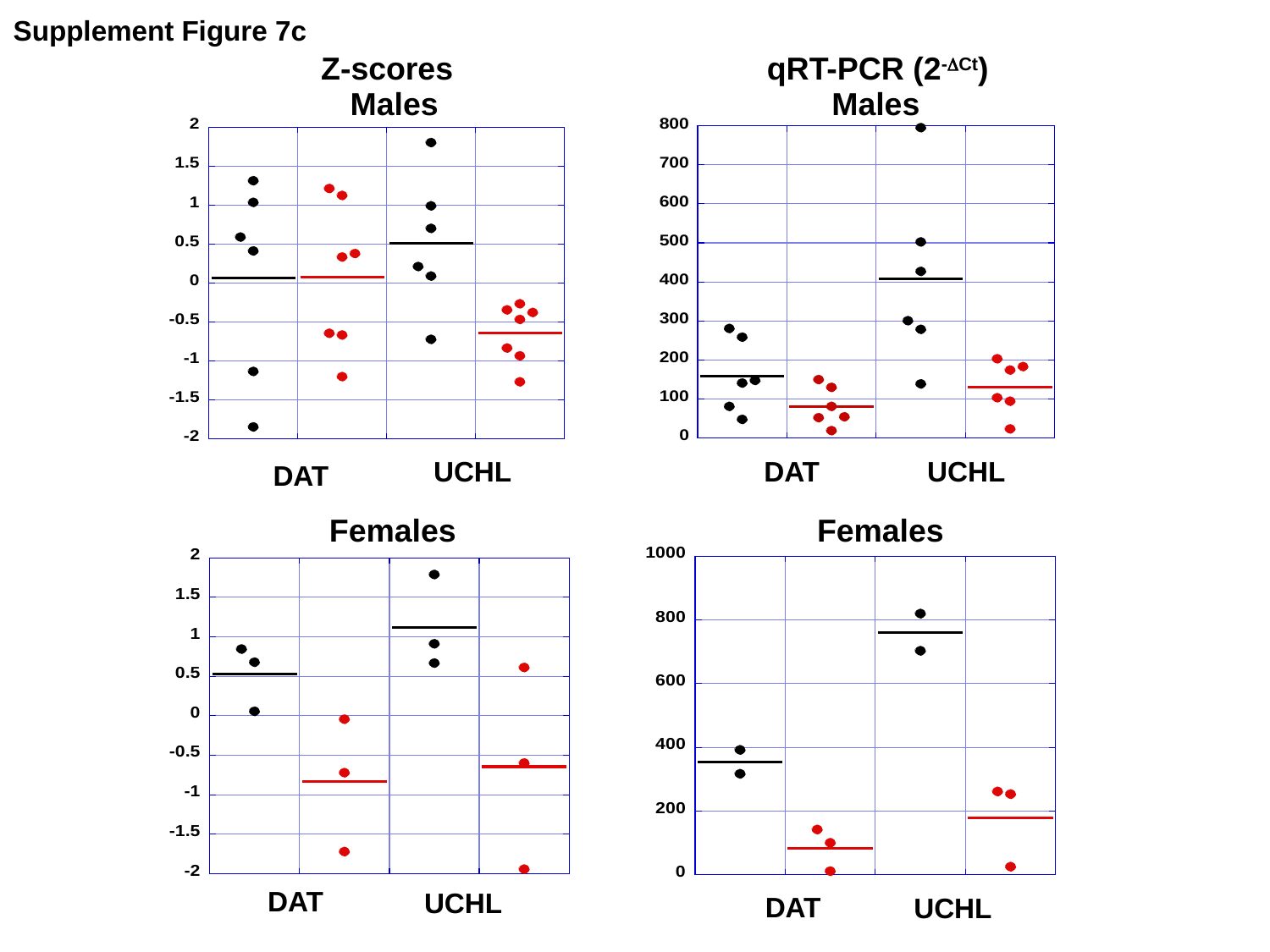

Supplement Figure 7c
Z-scores
qRT-PCR (2-ΔCt)
Males
Males
UCHL
DAT
UCHL
DAT
Females
Females
DAT
UCHL
DAT
UCHL

## Slide 4
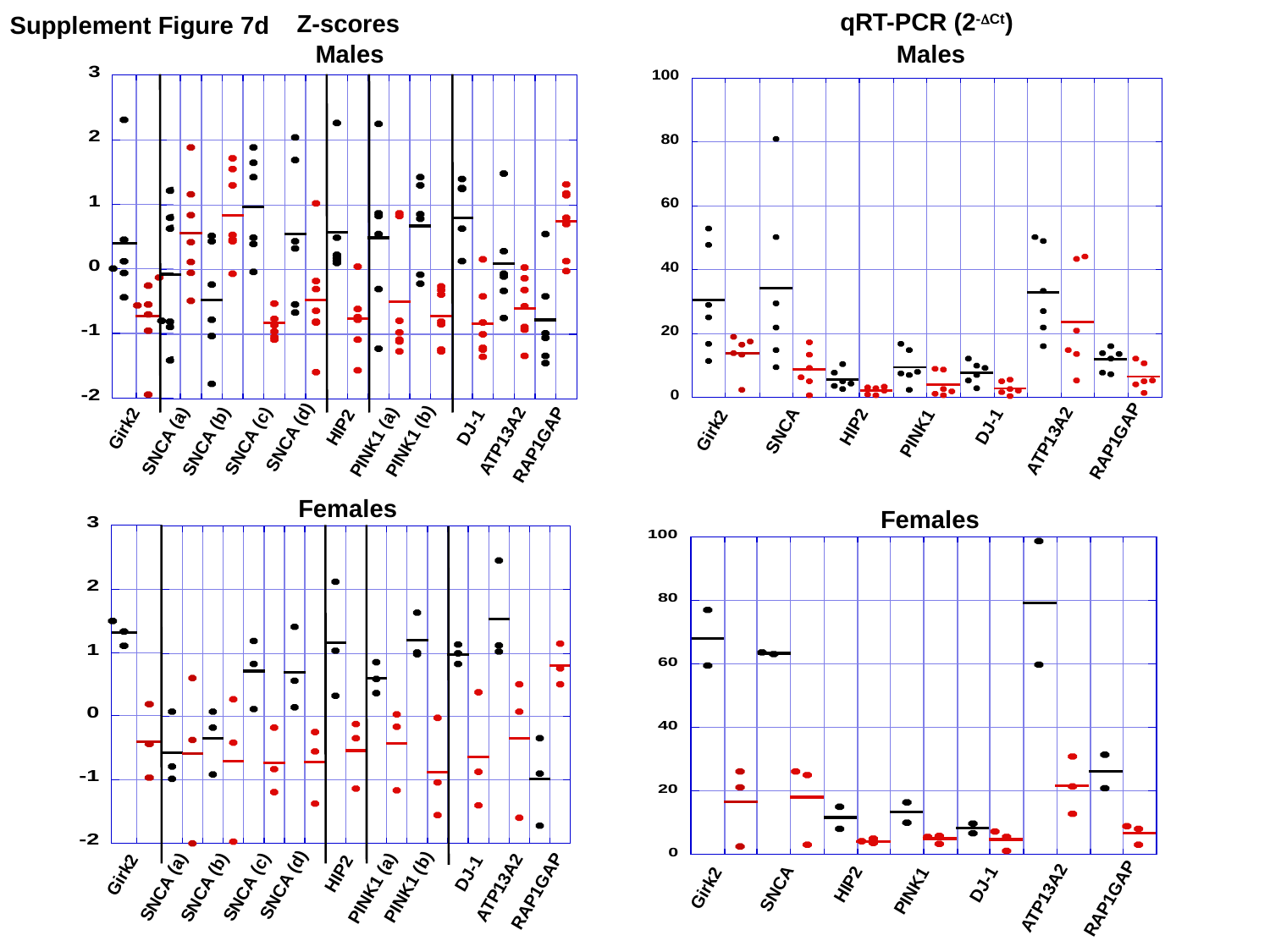

qRT-PCR (2-ΔCt)
Z-scores
Supplement Figure 7d
Males
Males
DJ-1
HIP2
DJ-1
HIP2
Girk2
Girk2
SNCA
PINK1
SNCA (d)
ATP13A2
PINK1 (b)
SNCA (a)
SNCA (c)
SNCA (b)
ATP13A2
RAP1GAP
PINK1 (a)
RAP1GAP
Females
Females
DJ-1
HIP2
Girk2
DJ-1
HIP2
SNCA (d)
PINK1 (b)
SNCA (a)
SNCA (c)
SNCA (b)
ATP13A2
PINK1 (a)
Girk2
SNCA
PINK1
RAP1GAP
ATP13A2
RAP1GAP

## Slide 5
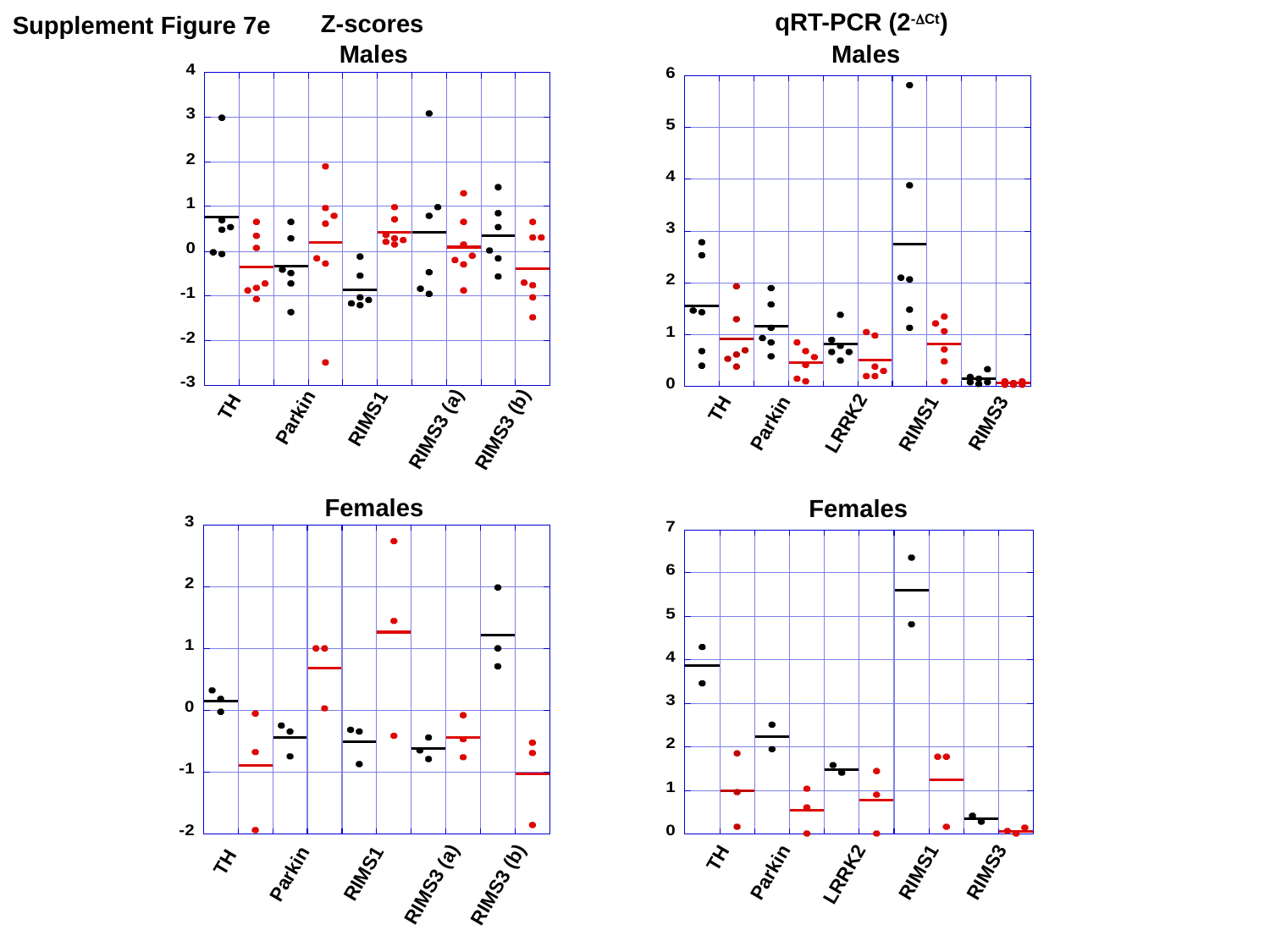

qRT-PCR (2-ΔCt)
Z-scores
Supplement Figure 7e
Males
Males
TH
TH
Parkin
RIMS1
Parkin
RIMS3
LRRK2
RIMS1
RIMS3 (a)
RIMS3 (b)
Females
Females
TH
TH
Parkin
RIMS1
RIMS3
RIMS1
Parkin
LRRK2
RIMS3 (a)
RIMS3 (b)
